# Genome-wide characterization of intergenic polyadenylation sites redefines gene spaces in *Arabidopsis thaliana*

**DOI:** 10.1186/s12864-015-1691-1

**Published:** 2015-07-09

**Authors:** Xiaohui Wu, Yong Zeng, Jinting Guan, Guoli Ji, Rongting Huang, Qingshun Q. Li

**Affiliations:** Department of Automation, Xiamen University, Xiamen, Fujian China; Key Laboratory of the Ministry of Education on Costal Wetland Ecosystems, College of the Environment and Ecology, Xiamen University, Xiamen, Fujian China; Graduate College of Biomedical Sciences, Western University of Health Sciences, Pomona, CA USA; Rice Research Institute, Fujian Academy of Agricultural Sciences, Fuzhou, Fujian China; Innovation Center for Cell Signaling Network, Xiamen University, Xiamen, Fujian China

**Keywords:** Alternative polyadenylation, RNA processing, Genome annotation, Intergenic region, 3’-UTR

## Abstract

**Background:**

Messenger RNA polyadenylation is an essential step for the maturation of most eukaryotic mRNAs. Accurate determination of poly(A) sites helps define the 3’-ends of genes, which is important for genome annotation and gene function research. Genomic studies have revealed the presence of poly(A) sites in intergenic regions, which may be attributed to 3’-UTR extensions and novel transcript units. However, there is no systematically evaluation of intergenic poly(A) sites in plants.

**Results:**

Approximately 16,000 intergenic poly(A) site clusters (IPAC) in *Arabidopsis thaliana* were discovered and evaluated at the whole genome level. Based on the distributions of distance from IPACs to nearby sense and antisense genes, these IPACs were classified into three categories. About 70 % of them were from previously unannotated 3’-UTR extensions to known genes, which would extend 6985 transcripts of TAIR10 genome annotation beyond their 3’-ends, with a mean extension of 134 nucleotides. 1317 IPACs were originated from novel intergenic transcripts, 37 of which were likely to be associated with protein coding transcripts. 2957 IPACs corresponded to antisense transcripts for genes on the reverse strand, which might affect 2265 protein coding genes and 39 non-protein-coding genes, including long non-coding RNA genes. The rest of IPACs could be originated from transcriptional read-through or gene mis-annotations.

**Conclusions:**

The identified IPACs corresponding to novel transcripts, 3’-UTR extensions, and antisense transcription should be incorporated into current Arabidopsis genome annotation. Comprehensive characterization of IPACs from this study provides insights of alternative polyadenylation and antisense transcription in plants.

**Electronic supplementary material:**

The online version of this article (doi:10.1186/s12864-015-1691-1) contains supplementary material, which is available to authorized users.

## Background

Polyadenylation is an essential process in which a poly(A) tail is added to the cleaved 3’-end of pre-mRNA [1]. The 3’ untranslated region (3’-UTR), via its embedded regulatory elements such as microRNA targets, plays an important role in mRNA post-transcriptional regulations [2]. Accurate determination of poly(A) sites helps to define the 3’-ends of genes, which is important for genome annotation and gene function studies. If a gene possesses more than one poly(A) site, it is subject to alternative polyadenylation (APA). Recent genomic studies have uncovered widespread occurrences of APA which can generate tremendous transcript diversity [2]. Majority of APA events occur within 3’-most exons, resulting in 3’-UTR shortening or lengthening and generate transcripts with different lengths [3]. It has been observed that global 3’-UTR shortening by APA can activate oncogenes in cancer cells [4], whereas 3’-UTR lengthening can occur during mouse embryonic development and differentiation [5, 6]. The average size of *Arabidopsis thaliana* 3’-UTRs in the latest TAIR10 annotation is 217 nt; the APA extension in the literature ranges from 25 % to 70 % [7–9]. How far are these estimates from reality? Several recent studies have uncovered widespread occurrences of APA sites in plants and algae, such as rice, Arabidopsis and *Chlamydomonas reinhardtii* [7, 9–12]. However, the study on the genome-wide evaluation of 3’-UTR extension in plants is scarce.

Whole genome tiling array and transcriptome sequencing studies have revealed the presence of unannotated genes in intergenic regions. 19–23 % of the Arabidopsis intergenic region was found to be transcribed using whole genome tiling arrays [13–15]. Hanada et al. identified more than 7000 small open reading frames with coding potential in the intergenic regions of the Arabidopsis genome [16]. Comparative analyses of three Brassicaceae species and six crucifer genomes have revealed approximately 90,000 conserved noncoding sequences that show evidence of transcriptional and post-transcriptional regulation [17]. Rose et al. predicted 336 novel multi-exon transcripts from human intergenic regions which were considered to be conserved during evolution [18]. LongSAGE tags of 15,892 from humans were found to be located in intergenic regions, many of which were generated from uncharacterized genes [19]. In addition, several recent genomic studies also emphasized the existence of 3’-UTR extensions in intergenic regions downstream of annotated genes. Lopez et al. used human ESTs (Expressed Sequence Tags) and observed a significant incidence of poly(A) sites lying in the 5–10 kb region past the stop codon and found as many as 5000 human genes with unreported 3’ extensions [20]. Several long transcripts spanning the whole poly(A)-poly(A) or stop-poly(A) distance were experimentally validated using a long-distance RT-PCR strategy [21]. Using comparative genomics and transcriptomics across vertebrates, Morgan et al. [22] found many conserved unannotated 3’ ends and reported several hundred novel 3’-UTR extensions. Using deep RNA-seq data, Miura et al. [3] found substantially distal novel 3’-UTRs generated by APA in human and mouse. Thousands of genes extend at least 500 nt past the most distal 3’ termini; some of these genes bear exceptionally long 3’-UTRs (>10 kb). A dataset of bovine skin containing a total of 10,884 unannotated transcripts was discovered, 1035 of them were located within a 1 kb distance to a nearby genes and only four potential protein coding transcripts were detected in intergenic regions [23].

Only limited studies laid emphasis on the intergenic regions and the 3’-UTR extensions in plants. Moghe et al. [24] found 6545 intergenic transcribed fragments (ITFs) in Arabidopsis, ~30 % of which are likely associated with annotated genes. Many of these ITFs may be background or noisy transcripts, whereas only 237 ITFs are likely originated from novel genes and 49 ITFs are with translation evidence. Using data from direct RNA sequencing (DRS), Sherstnev et al. [9] proposed the first 3’-UTR annotation for 165 Arabidopsis coding genes without annotated 3’-UTRs in TAIR10 and re-annotated the 3’-UTRs of 10,215 Arabidopsis genes. Duc et al. [25] used DRS to study poly(A) site choice in Arabidopsis *fpa* mutants and identified 170 intergenic regions differentially expressed in *fpa-7*. Schurch et al. [26] illustrated several examples of improved annotation of 3’-UTRs in human, chicken, and Arabidopsis by combination of DRS, RNA-Seq and ESTs. Till now, a large amount of poly(A) sites were found to be in intergenic regions in Arabidopsis [7, 9, 12]. These intergenic poly(A) sites might be associated with 3’-UTR extensions, novel genes, or antisense transcription [7, 20], whereas they remain poorly characterized. Although many pioneering works have been done for the improvement of 3’-UTR annotation in Arabidopsis, no attempt has been made to evaluate intergenic poly(A) sites systematically at the genome level in plants. Accurate reconstruction of parent transcripts and annotation of novel genes from these intergenic poly(A) sites remain challenging.

In this study, we set out to identify and evaluate a large number of intergenic poly(A) sites from poly(A)-tag sequencing to uncover thousands of previously unannotated 3’-UTR extensions to known genes and frequent polyadenylated transcripts from potential novel genes in Arabidopsis. Characterization of these intergenic poly(A) sites will provide insights into the mechanisms of alternative polyadenylation and antisense transcription in plants.

## Results

### Determination of intergenic poly(A) sites

To characterize Arabidopsis intergenic poly(A) sites at a genome level, we used three wild type (WT) datasets from paired-end sequencing in a previous study [12]. More than two million poly(A) tags (PAT) could be mapped to Arabidopsis TAIR10 genome, generating 253,719 cleavage/poly(A) sites. 3’-UTRs in TIAR10 genome annotation were extended by 50 nt to improve the “recovery” of PATs that are in the vicinity of authentic 3’-UTRs due to microheterogeneity. Cleavage sites in the same gene that are located within 24 nt of each other were then grouped into poly(A) site clusters (PACs) to eliminate the impact of microheterogeneity of nearby poly(A) sites [7]. Finally, more than 54,000 PACs are defined by these highly confident PATs, covering 16,834 genes. More than half (~52 %) of these PACs fall within 3’-UTRs defined by over 90 % of all PATs (Table 1). About 7 % and 4 % of PACs map to protein coding regions and introns, respectively. Only few PACs map to 5’-UTRs. Approximately 5 % of PACs fall within ambiguous regions of the annotated genome owing to alternative transcription or RNA processing. About 30 % of PACs map to putative intergenic regions, defined by ~3 % of all PATs. These intergenic PACs (IPACs) are located between two annotated genes on the same DNA strand and are retained for further analysis in this study. The number of IPACs according to the original TAIR10 annotation would have been larger if 3’-UTRs were not extended by 50 nt. This 50 nt regions are intergenic according to the original annotation by TAIR10. A total of 7037 PACs from 167,584 PATs lie within the boundaries of TAIR10 transcripts (in the extended 50 nt regions). These PACs are denoted as 3’-UTR PACs and thus are not included in the study of intergenic PACs.

**Table 1:** Genomic distribution of PACs and PATs

Region	PAC#	PAT#	PAC%	PAT%
3’-UTR^a^	28,266	2,129,949	51.77 %	91.13 %
Intergenic	16,567	64,550	30.34 %	2.76 %
CDS	3773	10,944	6.91 %	0.47 %
Intron	2301	6744	4.21 %	0.29 %
5’-UTR	208	6567	0.38 %	0.28 %
Exon	406	2791	0.74 %	0.12 %
Pseudogenic exon	102	1193	0.19 %	0.05 %
AMB^b^	2976	114,436	5.45 %	4.90 %
Total	54,599	2,337,174	100.00 %	100.00 %

^a^3’-UTRs were extended by 50 nt beyond the annotated poly(A) site by TAIR10

^b^AMB: ambiguously mapped at regions that have different annotations due to alternative transcription or RNA processing

### Distribution of IPACs

**Fig. 1:**
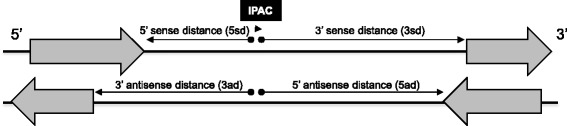
Schema of distances from an IPAC to its nearby genes. Solid lines represent DNA sequences; shaded arrows indicate genes and their transcription directions

To characterize the IPACs, we first calculated distances from each IPAC to its 5’ sense gene, 3’ sense gene, 5’ antisense gene, and 3’ antisense gene, respectively (Fig. 1). The median distance from IPACs to the upstream 5’ sense genes is only 122 nt even though 3’-UTRs were already extended by 50 nt (Additional file 1: Figure S1). This is much shorter than that to 3’ sense or antisense genes (2237 to 5165 nt), indicating that IPACs tend to be closer to upstream 5’ sense genes. This is not surprising because there may be a promoter region before the next gene at the 3’-end of the IPAC. What is surprising, however, is the average distance from IPACs to 5’ sense genes is 1629 nt (more than 13 folds than the median distance 122 nt), which indicates that distances from IPACs to 5’ sense genes are not distributed evenly.

The majority of IPACs are in the vicinity of the ends of upstream 5’ genes (Additional file 1: Figure S2A). IPACs also tend to be close to the starts of the downstream 3’ genes (Additional file 1: Figure S2B). This result indicates that IPACs are more likely to be distributed close to the neighboring genes especially the upstream 5’ gene and distribute sparely in the middle of the intergenic regions. Since different intergenic regions are in different lengths, to compare the relative positions of IPACs, we normalized the distances from IPACs to the 5’ genes by the lengths of the intergenic regions, and the result is shown in Additional file 1: Figure S2C. The majority of IPACs (8020 out of 16567, ~48 %) are located in the start (X-axis ≤ 0.1) of intergenic regions (Additional file 1: Figure S2C).

For IPACs whose antisense parts are also intergenic, we calculated their distances to the neighboring antisense genes. These IPACs are not as close to the 5’ or 3’ antisense gene as the sense genes, though they are also close to neighboring genes (Additional file 1: Figures S2D, E, and F). This result indicates that antisense genes have much less impact on IPACs. In other words, IPACs are much more likely to be originated from the sense genes rather than the antisense genes. Interestingly, IPACs are more likely located near 3’ antisense genes, which is consistent with the observation that IPACs are closer to sense 5’ genes than 3’ genes. As shown in Additional file 1: Figure S2F, ~20 % (1988 of 16,567) IPACs are located within the first 10 % of the intergenic regions, whereas another spike was also observed at the end of the intergenic regions, indicating that IPACs tend to be distributed near both the 5’ or 3’ antisense genes rather than the middle of intergenic regions.

To estimate background noise, we randomly selected 16,567 nucleotide positions (as to mimic the poly(A) sites; the same number as total IPACs) in intergenic regions. Distances from these random positions to neighbouring sense and antisense genes were calculated as background distances. As shown in Additional file 1: Figure S3, there is a background slope, which may be due to the finite length of intergenic regions in the genome. This background slope was also observed in a previous study [20]. Because a randomly selected position cannot be located at an arbitrary long distance from the nearest gene, the distribution of random positions is slightly skewed toward shorter distances. This slope is greatly reduced after normalizing the distance by the length of the respective intergenic regions (Additional file 1: Figures S3C and F). The distribution of distance from IPACs to 5’ sense genes is significantly different from the background distribution (Additional file 1: Figure S2A vs. Additional file 1: Figure S3A). The distribution of distance from IPACs to 3’ antisense genes seems also very different from the background distribution (Additional file 1: Figure S2E vs. Additional file 1: Figure S3E). Our results indicated that distributions of PACs are closer to the upstream or downstream sense genes.

### Classification of IPACs

**Fig. 2:**
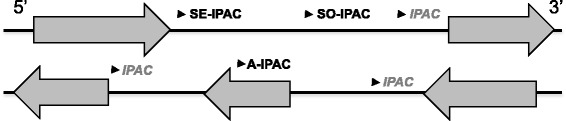
Schema of different classes of IPACs. SE-IPAC, IPAC in the 3’-UTR extension of its 5’ sense gene (Sense-Extension); A-IPAC, IPAC that is located in another antisense gene (Antisense); SO-IPAC, IPAC that is far from all nearby genes (Sense-Orphan)

Based on the distributions of the distances from IPACs to nearby sense and antisense genes, we classified IPACs into three categories (Fig. 2): SE-IPAC, IPAC in the 3’-UTR extension of its 5’ sense gene (Sense-Extension); A-IPAC, IPAC that is located in another antisense gene (Antisense); SO-IPAC, IPAC that is far from all nearby genes (Sense-Orphan). We focused on these three main cases of IPACs, the rest of the IPACs which may be close to the promoter of the 5’ sense gene (344) or the 5’ and 3’ antisense gene (791) were not analyzed in this study due to insignificance in numbers.

**Fig. 3:**
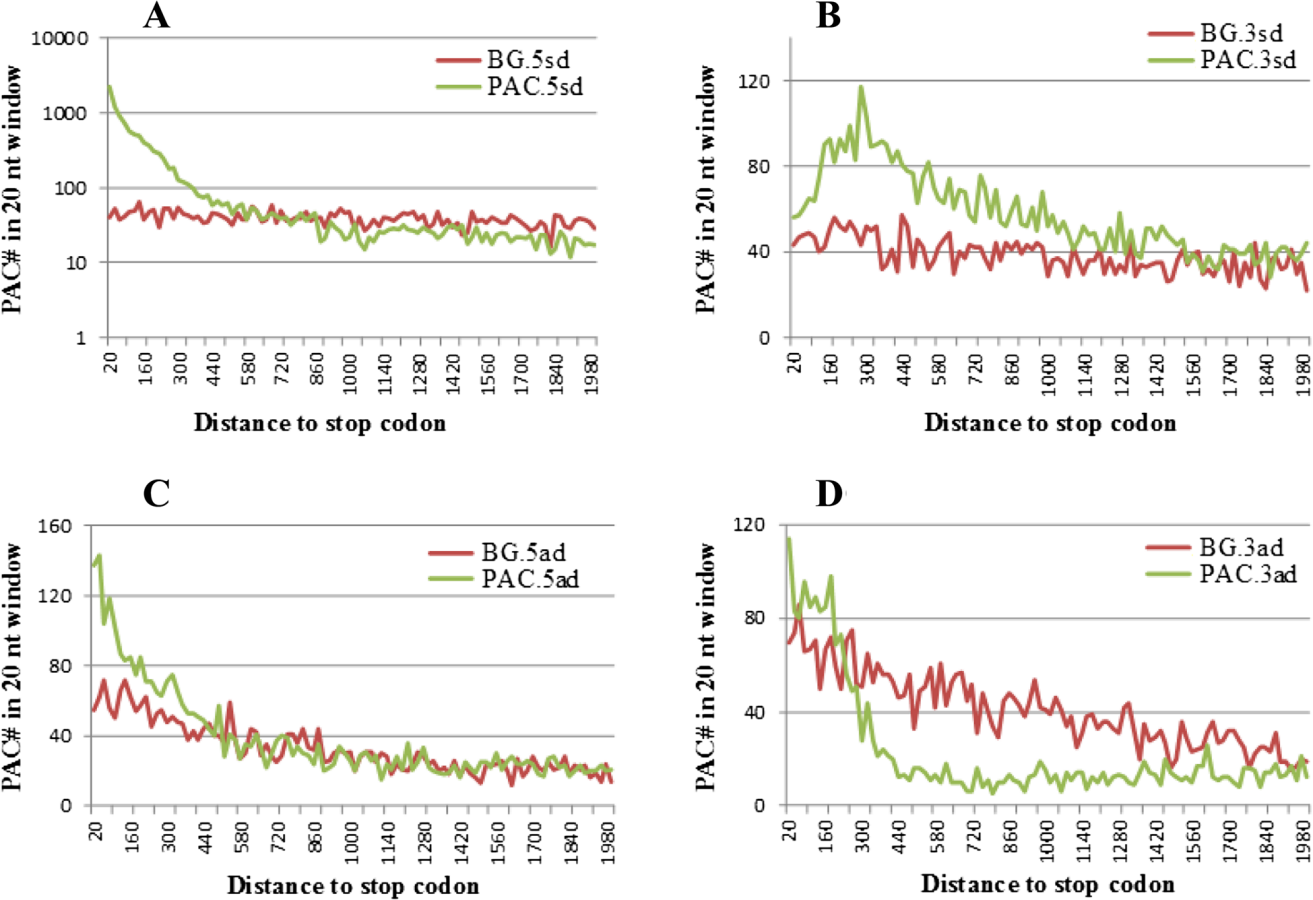
Distributions of distances from IPACs to neighboring sense and antisense genes using background distributions as baselines. Distances from IPACs or background nucleotide positions to 5’ sense gene (**a**), 3’ sense genes (**b**), 5’ antisense genes (**c**), and 3’ antisense genes (**d**). The curves in (**a**) are shown on a log scale

To determine an appropriate distance for SE-IPACs, we first calculated the number of IPACs and random background positions at increasing distance from the nearest annotated stop codon by intervals of 20 nt (Fig. 3a). The overall distributions of IPACs and background positions differ dramatically in the first 500 to 700 nts where the position at which IPACs disappearing over background becomes apparent. To identify this position more precisely, we computed the ratio of background positions to the total number of positions at a given distance as the false discovery rate (FDR). When the distance to the stop codon is 700 nt, the number of background positions within the 700 nt region is 1528 (FDR = 0.092); the number of IPACs is 10060. It is apparent from Fig. 3a that the distribution of IPACs becomes flatter at 700 nt past the stop codon. Therefore, given a FDR < 0.1 (0.092), 10060 IPACs are considered to be associated with events of 3’-UTR extension. These IPACs are classified as SE-IPAC (Sense Extension; Fig. 2). This result suggests that SE-IPACs may extend to 700 nt past the stop codon, which is considerably longer than the annotated 3’-UTR size [7]. It should be noted that the curves in Fig. 3a are shown in a log scale and the actual number of sites beyond 700 nt is very small relative to sites in the 0–700 nt range. In contrast, the extended 700 bp length in Arabidopsis is dramatically lower than the corresponding value observed from vertebrates (~9-12 kb) [20], which probably due to the more compact genome of Arabidopsis. For instance, the average size of human 3’-UTRs is ~1000 nt [20], while Arabidopsis only has a mean 3’-UTR size of 218 nt according to TAIR 10 annotation.

**Fig. 4:**
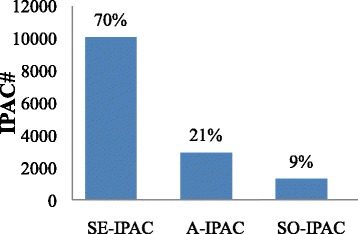
Statistics of three categories of IPACs

There are 4051 IPACs classified as antisense IPACs (A-IPAC) which may be from the transcripts of the antisense genes [7]. To further remove possible internally primed artifacts from poly(A)-tag sequencing, 1094 poly(A) sites with A/G rich in the downstream 20 nt were discarded. Although some genuine poly(A) sites might be removed from this step, we preferred a conserved estimation using the curated A-IPACs. Additional analyses of these A-IPACs are carried out in the next section. Excluding SE-IPACs and A-IPACs, there are 6053 IPACs located at 700 nt or more beyond the upstream stop codon. Distances from these IPACs to their nearby genes were then calculated for further characterization. The distributions of distances from IPACs to 3’ sense genes or 5’ antisense genes resemble the background curves (Fig. 3b and c; note that the scale is different from Fig. 3a). In contrast, distribution of IPACs to 3’ antisense genes shows considerably different from the background (Fig. 3d). Thus, for further characterization of this special group, these IPACs are defined as SO-IPACs, excluding those close to their 3’ antisense genes or 3’ sense genes (<700 nt). The three classes of IPACs are listed in Fig. 4. The majority (70 %) of IPACs belong to the SE-IPAC group, suggesting that most IPACs may be associated with 3’-UTR extension.

### Characteristics of different classes of IPACs

#### General sequence characteristics of IPACs

**Fig. 5:**
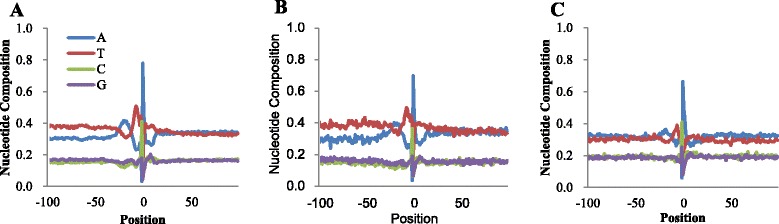
Position-by-position base composition of IPACs in different classes. (**a**) SE-IPAC; (**b**) SO-IPAC; (**c**) A-IPAC

To gain further insight into IPACs of different classes, the relative base composition of the sequences surrounding IPACs was studied. Such analyses have been thought to be an effective way to identify important sequence trends and probable poly(A) signals [7, 12, 27]. Results show that base compositions surrounding SE-IPACs and SO-IPACs are similar (Fig. 5a and b) but with less A/T difference in the NUE (near upstream element) region of SO-IPACs. This difference probably reflects distinct poly(A) signals used in different classes of IPACs. Particularly, the profile of SE-IPACs is indistinguishable from that of 3’-UTRs PACs [7, 27], reflecting that these SE-IPACs are very probably associated with known 3’-UTRs. These SE-IPACs that possess the general poly(A) signals may derive from genuine alternative polyadenylation, unannotated genes, or transposons [25]. In contrast, the profile of A-IPACs seems to be different (Fig. 5c). There is little preference for T across the whole profile, but there is an increased occurrence of A throughout the entire region. We further divided A-IPACs into four groups according to their antisense regions and found that A-IPACs with different antisense regions show distinct profiles (Additional file 1: Figure S4). Therefore, the different profile of A-IPACs from that of SE-IPACs or SO-IPACs probably because that A-IPACs are located in the antisense parts of annotated genes, sharing the same nucleotide base compositions as their complementary parts.

We then examined the frequency of the use of poly(A) signal hexamers for different classes of IPACs. Poly(A) signal hexamers can be classified into three types: AATAAA, 1 nt variants of AATAAA (1 nt-variants), and no poly(A) signal (noPAS) (Additional file 1: Figure S5). As demonstrated in many genetic assays, the most dominant poly(A) signal for promoting polyadenylation in plants is AATAAA. Approximately 7 % and 34 % of SE-IPACs and SO-IPACs have AATAAA and its variants, which resembles the observation from 3’-UTR PACs. This reflects that SE-IPACs and SO-IPACs may share similar poly(A) signals with 3’-UTR PACs [27]. In contrast, A-IPACs are associated with weaker (or different) signals than other groups of IPACs. There are only 3 % and 23 % of AATAAA and 1 nt variants for A-IPACs, which are much lower than other classes. Thus, the strength of poly(A) signal of A-IPACs is different from other types of IPACs, suggesting distinct mechanisms in the use of these signals.

Our next objective was to obtain a measure of the accuracy of these IPACs. Such a measure would be useful in the selection of the most promising candidates for experimental validation of novel 3’-UTRs or intergenic transcripts. We adopted the poly(A) site prediction tool for Arabidopsis called PASS [28] to predict IPACs. First, the sequence of upstream 300 nt and downstream 100 nt of each IPAC was trimmed. Given a sequence, the output of PASS is a series of scores of each position of this sequence. The bigger a score is, the higher probability of this position being a real poly(A) site. Using the default parameters of PASS, we calculated the scores for all IPACs. Moreover, randomly selected 2000 3’-UTR PACs and background positions were used as controls. The output scores for each position of the 400 nt sequences of each group were averaged. As can be seen from Additional file 1: Figure S6, the score profiles of IPACs and 3’-UTR PACs are highly distinguishable from that of background positions. We observed a local peak in the position of poly(A) site (300 nt) with a sharp decline around the 3’-UTR PACs or IPACs, suggesting that these IPACs share similar sequence characteristics with 3’-UTR PACs.

### 3’-extensions of gene space from SE-IPACs

The majority of IPACs are in the case of SE-IPAC. A total of 6984 genes have 10060 SE-IPACs in their extended 3’-UTR regions. These genes play roles in functions like phosphorylation and cell recognition (Additional file 2: Table S1), which is suggestive of distinctive modes of actions and functions for SE-IPACs. 2762 SE-IPACs were supported by RNA-seq data, which were located within 200 nt of genes identified by Tophat/Cufflinks pipeline. Of the 6984 genes, there are 2035 genes that do not have any 3’-UTR PAC but have SE-IPACs in their extended downstream regions. There are 3255 genes that have more than two PACs in their 3’-UTRs, and 2913 extended 3’-UTR regions have more than two SE-IPACs. A previous study [9] discovered 165 coding genes with newly annotated 3’ UTRs and 429 genes with 3’ UTR elongation more than 50 nt (as the 3’ UTR was extended 50 nt in our study), 135 (82 %) and 335 (78 %) of these genes also possess SE-IPACs, reflecting the authenticity of the SE-IPACs discovered in this study. These SE-IPACs could be a valuable addition to the improvement of the Arabidopsis genome annotation. Considering these SE-IPACs as additional 3’-UTR PACs, there are up to 38,242 PACs in the 3’-UTRs covering 17,575 genes. These SE-IPACs supply poly(A) sites for 2736 genes that were without an annotated poly(A) site in TAIR10 (SE-IPAC lists are provided in Additional file 3).

**Fig. 6:**
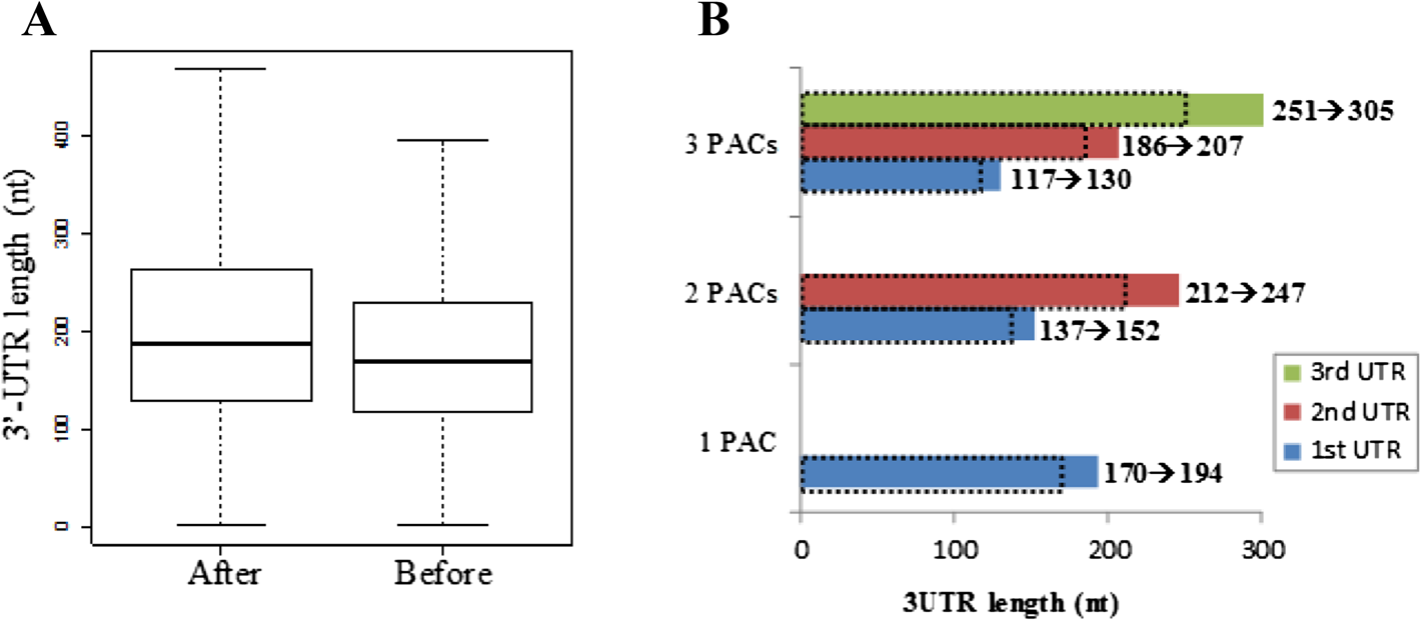
Changes in 3’-UTR lengths after including SE-IPACs. (**a**) Changes in 3’-UTR lengths using all genes with PACs; median lengths are 188 nt and 169 nt. (**b**) Change of median 3’-UTR lengths in function of the number of PACs after extending. Genes with one, two, and three PACs were selected to calculate the 3’-UTR length of each individual PAC. The histogram “3PACs” uses genes with 3 PACs and shows the median 3’-UTR length of the first (blue), second (red), and third PAC (green), respectively. The dashed box denotes the 3’-UTR length before extending. The solid box denotes the 3’-UTR length after extending. Numbers denote the 3’-UTR lengths before and after extending

To assess the changes in 3’-UTR lengths after recruiting SE-IPACs as 3’-UTR PACs, we calculated 3’-UTR lengths using original 3’-UTR PACs and the expanded ones. As shown in Fig. 6a, the length of 3’-UTR after extending is significantly longer than before extending (Wilcoxon test, *P value* = 9.39e-178). The median length of extended 3’-UTRs is 188 nt, which is 19 nt longer than the original 3’-UTR length (169 nt; using all 3’-UTR PACs). The difference of the mean length is even larger. The mean length of extended 3’-UTRs is 217 nt, which is 34 nt longer than the original 3’-UTR length (183 nt; using all 3’-UTR PACs).

We next assessed the change of 3’-UTR length as a function of the number of observed 3’-UTR PACs (Fig. 6b). In genes with three PACs after extending, the average 3’-UTR lengths of the first, second, and third PACs are 130, 207, and 305 nt, respectively. It is suggested that multiple PACs on the 3’-UTR are interspersed on average every 80 to 100 nt. These lengths after extending are 13 nt, 21 nt, and 54 nt longer than those before extending. For genes with two PACs, the 3’-UTR lengths after extending are 15 nt and 35 nt longer than those before extending. For genes with single PAC, the 3’-UTR length after extending is 24 nt longer than that before extending. This result also suggests that the 3’-UTR length of distal PACs tends to be lengthened to a larger extent than that of proximal ones.

### Potential novel transcripts from SO-IPACs

There are 1317 SO-IPACs which are located far from nearby genes (Fig. 4). These SO-IPACs are probably from novel transcripts. Compared to SE-IPACs, there are a lot less SO-IPACs, which may be due to that the genome annotation of Arabidopsis is quite complete and there are not many novel genes to be discovered. To validate these SO-IPACs, we employed five public RNA-seq datasets [29–33]. Novel genes not annotated in TAIR10 genome annotation were discovered from these RNA-seq datasets using the Tophat/Cufflinks pipeline [34]. Total 161 SO-IPACs were located within novel genes identified from RNA-seq data. If a 200 nt margin is allowed for comparison, 196 SO-IPACs were validated. How many uncharacterized genes may be originated from these SO-IPACs? An upper boundary for the number of transcripts detected in our analyses would be the 1317 identified SO-IPACs from unannotated intergenic regions. This is likely to be an overestimate, as multiple PACs may be derived from the same gene because of alternative polyadenylation. An estimation that includes this possibility may be obtained by evaluating the relative distance of PACs within the annotated genes and considering clusters of nearby IPACs derived from the same gene. Here we estimated a more likely number of potential novel genes. Previous studies have shown that APA is widespread in various species [2, 7, 35–37], we first estimated the distance of two PACs within 3’-UTR. Only 3’-UTR is used because the majority of PACs are in this region. The average length of 3’-UTR region after extending is 935 nt. The average number of PACs in the extended 3’-UTRs is 2.17. Therefore, the estimated average distance between two PACs in a gene is 431 nt. This distance is used to cluster SO-IPACs to estimate the number of potential genes. 592 out of 1317 SO-IPACs are located within a distance of 2000 nt of their nearby genes based on TAIR 10 annotation. At this distance, it is unlikely that a complete gene is inserted between IPAC and the upstream annotated gene, as Arabidopsis protein coding genes are on average 2343 nt long and separated by 5086 nt according to TAIR10 annotation. Even if exceptions are possible, we consider these IPACs to be pertaining to the nearest annotated genes, or false positives. This step removed some genuine orphan sense IPACs, but we preferred this conservative approach to focus on IPACs of high confidence. The remaining 725 IPACs located 2000 nt past their nearby genes were selected for further estimation of the potential novel genes. After clustering, there are 598 clusters, and the maximum number of SO-IPACs within a cluster is 8. Next, we used ESTs or RNA-seq data to verify these clusters. If the dominant IPAC in a cluster is located within the 500 nt of a given EST or within 200 nt of the novel genes discovered from RNA-seq, then it is considered as a of a potential transcript. Using this approach, 127 and 171 clusters can be verified by RNA-seq data and ESTs, respectively. Therefore, one would obtain 127 to 171 potential novel genes from these SO-IPACs as a lower boundary. These clusters contain 136 to 220 IPACs; the maximum number of IPACs in a cluster is three.

**Fig. 7:**
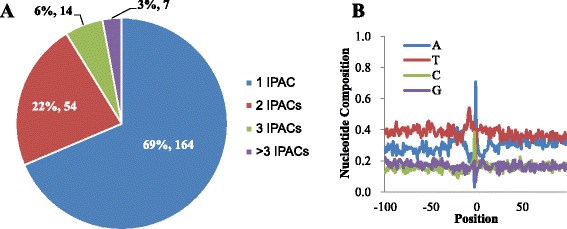
SO-IPACs associated with lncRNAs. (**a**) Statistics of lncRNA genes with different number of SO-IPACs; (**b**) single nucleotide compositions of SO-PACs located in lncRNAs

What is the coding potential of the novel genes associated with SO-IPACs? First we estimated the possible protein coding regions from these SO-IPACs. The average length of 3’-UTR from all PACs in 3’-UTRs is 200 nt. We trimmed upstream 500 nt to 200 nt region from each SO-IPAC to estimate the coding potential. Using the coding-potential calculator (CPC) [38] to calculate the coding potential of these 300 nt sequences, 37 SO-IPACs were considered as coding. We also utilized 6545 ITFs from previous study [24] to assess whether these SO-IPACs are transcribed or not. 259 (20 %) SO-IPACs are located within 100 nt of the ITFs, suggesting the transcription possibility of these IPACs. However, the relatively low percentage of IPACs overlapping with ITFs may be due to that most ITFs are significantly shorter and expressed at significantly lower levels [24] and thus cannot be detected by our sequencing data. Together, these results indicate that a small fraction (less than 20 %) of these SO-IPACs may be associated with protein coding genes, reflecting that the majority of them may be resulted from noncoding transcripts. The previous study [9] identified 132 snoRNAs (small nucleolar RNAs) that are located in intergenic regions defined in our study, 19 of them possess IPACs from this study, indicating that a small part of IPACs may be associated with non-coding RNAs. Next, we further used plant long non-coding RNAs (lncRNAs) [39] to search possible ncRNAs that embed SO-IPACs. Total 350 SO-IPACs could be mapped to 239 lncRNAs. The majority of these lncRNAs (164 from 239) possess only one SO-IPAC (Fig. 7a). 31 % of lncRNAs have more than one SO-IPAC, whereas the APA extent in animals is up to 66 % [37]. The single nucleotide composition profile of these SO-IPACs resembles that of all SO-IPACs (Fig. 7b vs. Fig. 5b), indicating that no different sequence characteristics may be used by PACs in lncRNA.

The previous study [40] predicted more than 33,000 open reading frames (ORFs) encoding peptides in Arabidopsis using a bioinformatics approach, 1044 of which were supported by several kinds of annotation data such as tiling hybridization experiments. As small genes encoding peptides are often overlooked in genome annotations [41], it raises the possibility that SO-IPACs may be associated with genes with small size. To test this possibility, we scanned the upstream regions of SO-IPACs for previously identified small ORFs with predicted small signal peptides (SSP) [40]. From the 1317 SO-IPACs, 651 (49 %) of them were located within 2000 nt downstream of small ORFs (the list of SO-IPACs associated with small ORFs is provided in Additional file 3). Up to 406 SO-IPACs can be associated with small ORFs that validated by at least one of the following five evidences, including the tiling experiments, the massively parallel signature sequencing (MPSS) dataset, the plant protein database, the orthologs in rice, and the single-linkage clustering. 103 SO-IPACs are associated with small ORFs that can be validated by at least two of the five evidences. As the poly(A) site marks the end of a gene, the presence of a poly(A) site might be considered as an evidence for the identification of small ORFs or SSPs.

### A-IPACs may be associated with antisense transcription

**Fig. 8:**
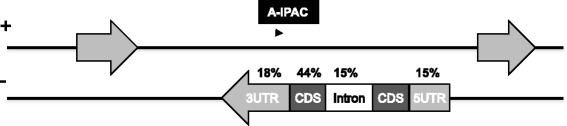
Distribution of A-IPACs whose antisense is not intergenic

There are 21 % (2957) A-IPACs whose antisense strand is not intergenic (Fig. 4). These IPACs may be originated from antisense transcripts. 1073 IPACs were supported by RNA-seq data, which were located within 200 nt of genes identified from RNA-seq data. 18 % of A-IPACs (539) are in the 3’-UTR region of its antisense gene. Interestingly, 44 % of them are from CDS, 15 % are from introns (Fig. 8). Majority of the antisense genes (91 %) of these A-IPACs are protein coding genes. These A-IPACs affect 2265 protein coding genes as well as 39 genes that are not protein coding; the latter include genes that encode 15 transposable element genes, 12 ncRNA, 8 pseudogenes, 3 miRNA, and 1 rRNA. Some interesting trends are apparent in the Gene Ontology analysis of these antisense genes (Additional file 2: Table S2). These genes involve in functions like response to abiotic stimulus, associated with hormonal responses and growth regulations, suggesting important roles for those genes with A-IPACs.

Antisense transcription may affect gene expression in both negative and positive strands [42]. We then inspected the expression levels of the A-IPACs and their respective targets to provide possible mechanisms of gene expression regulation. The number of PATs of the A-IPACs and the total number of PATs of their target genes were used as expression level analysis. There is a relatively low but significant correlation between the expression levels of A-IPACs and their targets (Pearson’s correlation = 0.11, *P value* = 1.977e-06). This result indicates that antisense transcription may serve to regulate the expression of target genes.

### Intergenic poly(A) sites in *oxt6* mutant

To examine whether the characteristic of intergenic poly(A) sites in other mutant is similar to that in WT, we studied the intergenic poly(A) sites in a mutant (*oxt6*) deficient in CPSF30 (30-kD subunit of the Cleavage and Polyadenylation Stimulatory Factor) expression [12]. Using the same strategy of classification of IPACs in WT, 9281 SE-IPACs, 1071 A-IPACs, and 1156 SO-IPACs were found in *oxt6* mutant (Additional file 1: Figure S7A). As expected, majority of the IPACs are SE-IPACs, while the number of A-IPACs in *oxt6* mutant is much lower than that of WT (Additional file 1: Figure s4). The relative base composition of the sequences surrounding SE-IPACs is similar to that of SO-IPACs (Additional file 1: Figures S7B and C), both of which are indistinguishable from that of 3’-UTRs PACs in *oxt6* mutant [12]. Again, this result reflects that SE-IPACs or SO-IPACs are very likely associated with known 3’-UTRs. In contrast, the profile of A-IPACs is slightly different from that of other IPACs (Additional file 1: Figure S7D), which is also observed in WT.

Next, we examined whether there is a large-scale shift of IPAC usage between *oxt6* mutant and WT. Up to 5283 (46 %) from the 11,508 IPACs in *oxt6* mutant are located within 50 nt of IPACs in WT, indicating that about half of poly(A) sites in intergenic regions are shared by both WT and *oxt6* mutant. The percentage of common IPACs between *oxt6* mutant and WT is lower than that of 3’-UTR PACs (74 %) [12] while higher than that of CDS or intron PACs (25 %), which may due to that IPACs could be originated from any genomic regions. In addition, there is a relative high and statistically significant correlation of the read counts of common IPACs between *oxt6* mutant and WT (Pearson correlation = 0.44, *P value* = 0), indicating that poly(A) site usage of common IPACs are unlikely altered in *oxt6* mutant. However, up to 54 % of IPACs in *oxt6* mutant are not present in WT, reflecting that a number of IPACs are specific for either the WT or mutant. Similar to the observation of IPACs, 3’-UTR poly(A) site choice in a large majority of Arabidopsis genes is also altered in *oxt6* mutant [12].

## Discussion

Based on a systematic investigation of 16,000 poly(A) sites in intergenic regions, the distribution and characteristics of these poly(A) sites were revealed. Such a whole genome level work extended the boundary of gene spaces by redefining the ends of transcription. Except for cases of A-IPACs whose antisenses are not intergenic, profiles and distributions of these IPACs are similar to those of 3’-UTR PACs, indicating that these IPACs share similar sequence charascteristics of general 3’-UTR PACs. This result was found in both WT and *oxt6* mutant. The majority of the IPACs (70 %, more than 10,000) are in the case of SE-IPAC, which would extend 6985 TAIR10 transcripts beyond their 3’-ends, with a mean extension of 134 nt. These thousands of confident IPACs add 5795 alternative 3’-UTRs to 4249 gene models with annotated 3’-UTRs and 4268 new 3’-UTRs to 2736 gene models without any annotated 3’-UTR. These 3’-UTRs extended 1.3 Mb of unannotated sequences (only the extended parts are counted) into gene space, and the average 3’-UTRs are 134 nt longer than the TAIR10 average of 217 nt.

SE-IPACs that extend the 3’-ends of known genes may result from several distinct mechanisms. First, these PACs can be the results of read-through transcriptions or alternative cleavage and polyadenylation, when the long-range PAC is located in the same 3’-exon as one or more other PACs. For example, 75 (44 %) from the 170 previously discovered intergenic read-throughs [25] also possess the SE-IPACs in this study. The second possible mechanism that may explain long-range PACs is alternative splicing, in which each alternative 3’-exons can possess its own PAC [43]. We excluded PACs in introns or CDS as much as possible in our analysis. However, since many splicing events may remain unreported, some long-range PACs may be originated from alternative splicing, especially the distal PACs [20]. The third possible mechanism is the presence of transcriptions that starts in the 3’-exons [44]. Some of these distal IPACs could correspond to partial, forward-strand transcripts, for which no function has been reported yet. An additional mechanism may be that the putative antisense transcripts corresponding to the IPAC of the associated sense transcript. For example, a SE-IPAC on chromosome 5 identified in this study is shared by At5g06290 and At5g06280, which was also reported in a previous study [45]. The intergenic region (351 bp) around this IPAC was found to direct GUS (β-Glucuronidase) expression in a mutually exclusive manner during Arabidopsis growth and development.

Our protocol identifies 1317 SO-IPACs corresponding to novel intergenic transcripts, or 9 % of the total IPACs. Two questions may be asked for these SO-IPACs. First, how many unannotated genes are actually present in the current genome? Verified by ESTs and RNA-seq data, 136 to 171 potential novel genes were estimated by clustering nearby SO-IPACs. 37 SO-IPACs are likely associated with protein coding transcripts. This is a relatively conserved estimation for the number of uncharacterized genes and protein coding genes in intergenic regions. This result is consistent with the extensive transcription observed in intergenic regions in a previous study where only four potential protein coding transcripts from more than 10,000 unannotated transcripts were found [23]. As many uncharacterized transcripts are expressed at low levels or in a tissue-specific manner, it is not expected that our analyses can detect all IPACs associated with these transcripts. Additional Arabidopsis tissues will have to be evaluated by RNA-seq or other experimental approaches to completely identify the compendium of transcripts that are encoded by the Arabidopsis genome. The availability of more sequencing data from different tissues will further provide opportunities to extend such comprehensive expression analyses. Second, what is the biologic function related to these SO-IPACs? Although it is possible that some of these SO-IPACs could result from spurious transcription, a large number of novel transcripts identified in intergenic regions have been demonstrated to correspond to bona fide genes which are differentially expressed and spliced [19]. The largest part of SO-IPAC diversity in the genome may represent nonfunctional sites that result either from background transcription or polyadenylation [20]. Previous studies found that intergenic transcripts accounted for more than half (57 %) of the predicted ncRNAs [46] and 66 % of long noncoding RNA genes have multiple PACs [37]. Additionally, noncoding sequences in Arabidopsis have been shown to participate in transcriptional and post-transcriptional regulations [17]. Some distal ncRNAs in intergenic regions can be reinterpreted as 3’-UTR extensions [3]. In this study, we found that 20 % SO-IPACs are associated with possible transcribed units and 27 % SO-IPACs are located in lncRNAs. Therefore, the majority of these SO-IPACs may represent ncRNAs or long-range 3’-UTR extensions of upstream known genes. Interestingly, 49 % of SO-IPACs can be associated with predicted small ORFs, with 106 (8 %) of them in the vicinity of small ORFs which can be validated by at least two kinds of annotation data, indicating that some SO-IPACs might be originated from genes encoding pepitides. Plant genes with small size are difficult to be predicted and largely overlooked [41], incorporation of SO-IPACs provided in this study would benefit the identification of genes encoding pepitide, ORFs or SSPs.

The intergenic region defined in this study is the region between two genes on the same DNA strand, but 2957 IPACs we identified corresponding to antisense transcripts for genes on the reverse strands (Fig. 4). These IPACs are of cases A-IPAC. Previous study in Arabidopsis was suggestive of wide-spread antisense transcription and a large number of PACs that were derived from antisense transcripts [7]. A-IPACs that are close to an adjacent gene oriented in sense strand may yield potential read-through transcripts extending to the respective IPAC [7]. A-IPACs that are far from any adjacent gene could not be associated with adjacent, convergently transcribed genes, which are remained to be investigated. Only 18 % of A-IPACs lie in the 3’-UTR region of its antisense gene, however the previous study [47] found that gene pairs with 3’ overlaps were significantly overrepresented among the cis-NAT-encoding (cis-natural sense antisense transcripts) genes. This discrepancy may due to that some of the A-IPACs may be originated from misannotation of the associated genes which remain further investegation. These A-IPACs affect a large number of protein coding genes (2265) and 39 other genes that are not protein coding, such as miRNAs, transposable elements, tRNA, snoRNA, etc. The correlation of expression levels between A-IPACs and their targets raises the possibility that antisense transcription may be associated with elevated gene expression [7]. However, strong linear correlations between the expression levels of genes pairs were rare and gene pairs may undergo discontinuous transcription and not be simultaneously transcribed even in the same tissue [13, 47], additional works are needed to further investigate the expression of A-IPACs and their antisense targets in more tissues or conditions.

Of the rest IPACs that are close to the 3’ or 5’-end of nearby antisense genes were not classified in this study, whereas they may be transcribed from the opposite strand with respect to the closest gene, which can play certain cis-regulatory roles in the regulation of neighbour genes [46]. 344 IPACs were found in the promoters of 279 3’ sense genes. As changing 5’-UTR boundaries can produce large changes in genes’ protein output [48], these IPACs could be associated with the mechanism of alternative transcription start site selection, which may lead to large differences in translation activity. However, given that polyadenylation typically does not occur near promoters [7, 49], the nature of these IPACs remains further investegation. Together, these unclassified IPACs may result from misannotation of the associated genes, extended read-through transcription, or as-yet unidentified transcripts.

In this study, it was observed that about half of IPACs in the *oxt6* mutant are not present in WT, suggesting that a number of IPACs are used in sample/condition specific manner. Another interesting question is then raised that how many IPACs would be conserved across the different plant species. In mammals, conservation of polyadenylation patterns has been studied extensively [50–52]. For instance, 4807 from 38,265 poly(A) sites were identified as conserved between human and mouse [50]. Approximately 10 % of human poly(A) sites were also detected in matched tissues in other four mammals [52]. In contrast, little is known about the conservation of poly(A) sites across diverse plant species. A recent study of APA sites between Arabidopsis and Medicago [53] revealed that 57 genes possessed conserved intronic poly(A) sites while 313 genes possessed conserved poly(A) sites in CDS. However, due to the relatively incomplete annotation of the Medicago genome and the limited source of poly(A) sites in other plant species, it is still challenging to conduct site-by-site comparative study of the conservation of IPACs across different plant species. Instead, here we employed highly conserved blocks from 20 Angiosperm Plant Genomes [54] to make a preliminary analysis of the conservation of IPACs. 222 to 2198 (19 to 43 %) of the three classes of IPACs were located in the conserved regions (Additional file 1: Figure S8A). If a 50 nt margin was allowed for each conserved region, the number of conserved SO-IPAC, A-IPAC, and SE-IPAC increased to 379, 2115, and 3673, respectively (Additional file 1: Figure S8B). More IPACs in class of A-IPAC were located in conserved regions than other two classes, suggesting a significant difference of the extent of conservation among these three classes (*χ*^2^ test, P value < 1.3e-131). Nevertheless, further comparative study needs to be carried out in the future to explore the conservation pattern of IPACs in plants when more poly(A) site data are available.

## Conclusions

The investigation of genome-wide intergenic poly(A) sites in this study provides further insight into the scope of plant transcriptomes. The detection of novel genes or alternative 3’-UTRs provides opportunities for functional studies of UTR-mediated gene regulation and the improvement of genome annotation. These neglected UTR regions and novel genes increase APA extent and transcript diversity, which should be incorporated in the search of regulatory elements such as microRNA targets. Our analyses help to add a large population of 3’-ends, expanding current Arabidopsis genome annotation. All together, these new annotations of 3’-UTR and intergenic units greatly revise the scope of post-transcriptional regulatory networks in plants. Still, genome annotation is a rather complex process, extensive manual curation will be needed to precisely annotate the whole genome.

## Methods

### Definition and identification of poly(A) sites

The datasets of WT and *oxt6* mutant were from previous studies [12]. The procedures for raw data processing have been described in detail elsewhere [7]. Briefly, raw reads were trimmed, filtered, and mapped to the Arabidopsis genome (TAIR10). Of note, in previous studies [7, 12], annotated 3’-UTRs in TIAR10 were extended by 120 nt and promoter regions were defined as the 2000 bp upstream of 5’-UTRs for the study of poly(A) sites in genic regions. However, here we focused on the analysis of intergenic poly(A) sites, therefore, 3’-UTRs were only extended by 50 nt to exclude intergenic PATs in the vicinity of annotated 3’-UTRs and promoter regions were not defined in this study. Mapped reads that represented possible internal priming by reverse transcriptase were removed from the subsequent analyses. Uniquely mapped reads that are located within 24 nt of each other were clustered to determine poly(A) clusters. Poly(A) clusters with three or more reads were used for further analysis. Poly(A) clusters were annotated using the latest Arabidopsis genome annotation (TAIR10).

### Classification of IPACs

Distances from each IPAC to its 5’ sense gene, 3’ sense gene, 5’ antisense gene and 3’ antisense gene were considered as the main metric to classify IPACs. The same number of positions in intergenic regions were randomly selected as background control to estimate FDR. Histograms (Additional file 1: Figures S2 and S3) were plotted to show the distributions of distances from IPACs or random intergenic positions to sense and antisense genes using an R package called ggplot2 [55] with 100 breakpoints. To determine an appropriate distance for SE-IPACs, the numbers of IPACs and random background positions were calculated from the nearest annotated stop codon to a maximum distance of 2000 nt by an interval of 20 nt (Fig. 3). FDR was defined as the ratio of background positions in a specific distance to the total number of background positions within the 2000 nt distance. SE-IPACs were first defined according to the distance from IPACs to their upstream genes. The distance that meets the criterion of FDR < 0.1 (700 nt) was obtained to differentiate SE-IPACs and other IPACs. IPACs that are located in their antisense genes were classified as A-IPACs. To further remove possible internally primed artifacts of A-IPACs, if the downstream 20 nt of a A-IPAC has more than 50 % of A or more than 60 % of A and G, then it is discarded. To determine the case of SO-IPACs, we first removed IPACs that are close to 3’ antisense genes using a similar strategy for SE-IPACs. When the distance to the stop codon is 200 nt, the FDR from background positions is 0.01. There are 791 IPACs located within the 200 nt region. These IPACs close to antisense promoter were removed. The rest IPACs of 5262 are either close to 3’ sense genes or far from all nearby genes. Setting the same distance cutoff as SE-IPACs, 344 IPACs are located within 700 nt from the corresponding 3’ sense gene. These IPACs close to sense promoter were also discarded for further analysis. The rest 1317 IPACs which are far from all nearby genes were classified as SO-IPACs.

### Data analysis

EST data were used for the conserved estimation of novel transcripts identified from SO-IPACs. EST/cDNA data were downloaded from TAIR website (ftp://ftp.arabidopsis.org/home/tair/Sequences/). These sequences were mapped to TAIR10 reference genome using GMAP [56]. The coordinates of 3’-ends of mapped ESTs were recorded. 180,805 aligned ESTs were located in intergenic regions. To verify SO-IPACs, the coordinates of SO-IPACs and EST 3’-ends were compared. If a SO-IPAC is located within 500 nt of any aligned EST, then it is considered as EST-verified IPAC.

Publicly available RNA-seq data [29–33] were also employed to validate SO-IPACs. For the data from Filichkin et al. [31], reads sequenced from normal physiological conditions were used and the 76 nt reads were truncated to 32 nt as recommended in [31]. For the data from [32, 33], we directly used the TopHat alignment files available at www.compbio.dundee.ac.uk/polyADB/ [25]. We ran TopHat [34] on each source of RNA-seq data using the following parameters: no multi-hits (−g 1), minimum anchor length 10 (−a 10), and minimum and maximum intron length 40 and 5000, respectively. Next, each alignment output from TopHat was assembled into putative transcripts by Cufflinks using TAIR10 genome annotation as the reference [34]. Then all Cufflinks assemblies were merged together using Cuffmerge [34] and genes in the final assembly denoted by “CUFF” and not overlapped with any annotated gene model in TAIR 10 were considered as novel genes. If a SO-IPAC is located within the novel genes identified from the TopHat/Cufflinks pipeline, then it is validated by the RNA-seq data. Because both the SE-IPAC and A-IPAC were associated with known gene models, we used genes in the Cufflinks assembly denoted by “CUFF” with FKPM (fragments per kilobase of exon per million fragments mapped) >0 to verify these two classes of IPACs.

Highly conserved blocks from 20 Angiosperm Plant Genomes [54] were employed to study the conservation of IPACs across plant species. Conserved regions of the mostCons set were downloaded from the Arabidopsis genome browser (http://genome.genetics.rutgers.edu/). Coordinates of IPACs were compared with the start/end coordinates of the conserved regions to examine whether an IPAC is located in a conserved region or not.

Gene functional annotation was performed using the DAVID Bioinformatics Resources [57]. For this, lists of genes associated with SE-IPACs and A-IPACs were compared with the list of all genes that are represented in the PAC datasets. Category designations: GOTERM_BP_FAT: Gene Ontology Biological Process classes; GOTERM_MF_FAT: Gene Ontology Molecular Function classes; GOTERM_CC_FAT: Gene Ontology Cell Component classes.

To estimate the protein coding potential of SO-IPACs in our dataset, we applied the Coding Potential Calculator (CPC, [38]) scanning for evidence for protein coding capacity. The input is the dataset of sequences of upstream 500 nt to 200 nt of all SO-IPACs. The output is the CPC score for each input sequence. Wilcoxon rank sum test for 3’-UTR lengths between two groups was carried out by the function wilcox.test in program R (http://www.r-project.org). To test the relation between SO-IPAC and ORF or SSP, the predicted small ORFs were downloaded from http://peptidome.missouri.edu [40]. Because the coordinates of these ORFs were based on TAIR6 annotation, we remapped DNA sequences of the ORFs to the TAIR10 genome using Bowtie2 [15] to obtain the updated coordinates. If a SO-IPAC is located within downstream 2000 nt of an ORF, then it is considered as associated with the ORF. To examine the correlation of expression level between A-IPAC and its target antisense gene, the total number of PATs of sense A-IPACs and targeted antisense genes were considered as expression levels. Test for correlation between paired samples based on Pearson's product moment correlation coefficient was performed by the function cor.test in program R. The occurrences of AATAAA and its variants in a studied region of IPACs were counted using a script written in Perl.

### Availability of supporting data

The data sets supporting the results of this article are included within the article and its additional files.

## Additional files

Additional file 1:
**Supplemental Figures: This file contains all the Supplemental Figures.**
Additional file 2:
**Tables S1 and S2.** This file contains all the Supplemental Tables including functional classes over-represented in the set of genes associated with SE-IPACs and A-IPACs. Additional file 3:
**Spread sheets with summaries of data compiled and used for the analyses in this study.**

## Abbreviations

IPAC: Intergenic poly(A) site clusters
3’-UTR: 3’ untranslated region
APA: Alternative polyadenylation
EST: Expressed sequence tags
ITF: Intergenic transcribed fragment
DRS: Direct RNA sequencing
WT: Wild type
PAT: Poly(A) tag
SE: Sense-extension
SO: Sense-orphan
FDR: False discovery rate
NUE: Near upstream element
CPC: Coding-potential calculator
snoRNA: Small nucleolar RNA
lncRNA: Long non-coding RNA
ORF: Open reading frame
SSP: Small signal peptide
MPSS: Massively parallel signature sequencing
CPSF30: 30-kD subunit of the Cleavage and Polyadenylation Stimulatory Factor
NAT: Natural sense antisense
FKPM: Fragments per kilobase of exon per million fragments mapped
