# Supplementary material for: Genome-wide characterization of intergenic polyadenylation sites redefines gene spaces in Arabidopsis thaliana
Source: BMC Genomics. 2015 Jul 9;16(1):511. doi: 10.1186/s12864-015-1691-1 (PMC4568572; doi:10.1186/s12864-015-1691-1)
Supplement: Additional file 3: — Spread sheets with summaries of data compiled and used for the analyses in this study. [file 12864_2015_1691_MOESM3_ESM.docx]

# Supplemental Tables

Table S1. Functional classes over-represented in the set of genes associated with SE-IPACs

| Category | Term | PValue |
| --- | --- | --- |
| GOTERM_BP_FAT | GO:0006468~protein amino acid phosphorylation | 1.40E-09 |
| GOTERM_BP_FAT | GO:0016310~phosphorylation | 3.79E-08 |
| GOTERM_BP_FAT | GO:0009875~pollen-pistil interaction | 1.37E-07 |
| GOTERM_BP_FAT | GO:0008037~cell recognition | 2.99E-07 |
| GOTERM_BP_FAT | GO:0048544~recognition of pollen | 2.99E-07 |
| GOTERM_BP_FAT | GO:0006915~apoptosis | 5.56E-07 |
| GOTERM_BP_FAT | GO:0006796~phosphate metabolic process | 7.47E-07 |
| GOTERM_BP_FAT | GO:0006793~phosphorus metabolic process | 8.06E-07 |
| GOTERM_BP_FAT | GO:0048610~reproductive cellular process | 5.65E-05 |
| GOTERM_BP_FAT | GO:0012501~programmed cell death | 2.03E-04 |
| GOTERM_BP_FAT | GO:0009856~pollination | 6.49E-04 |
| GOTERM_BP_FAT | GO:0045449~regulation of transcription | 8.10E-04 |
| GOTERM_BP_FAT | GO:0016265~death | 0.001169 |
| GOTERM_BP_FAT | GO:0008219~cell death | 0.001169 |
| GOTERM_BP_FAT | GO:0031640~killing of cells of another organism | 0.001983 |
| GOTERM_BP_FAT | GO:0001906~cell killing | 0.001983 |
| GOTERM_BP_FAT | GO:0030422~RNA interference, production of siRNA | 0.003251 |
| GOTERM_BP_FAT | GO:0051252~regulation of RNA metabolic process | 0.004549 |
| GOTERM_BP_FAT | GO:0006355~regulation of transcription, DNA-dependent | 0.004737 |
| GOTERM_BP_FAT | GO:0006874~cellular calcium ion homeostasis | 0.004882 |
| GOTERM_BP_FAT | GO:0055074~calcium ion homeostasis | 0.004882 |
| GOTERM_BP_FAT | GO:0006952~defense response | 0.006607 |
| GOTERM_BP_FAT | GO:0006855~multidrug transport | 0.008102 |
| GOTERM_BP_FAT | GO:0007047~cell wall organization | 0.009333 |
| GOTERM_BP_FAT | GO:0031050~dsRNA fragmentation | 0.00988 |
| GOTERM_BP_FAT | GO:0043331~response to dsRNA | 0.00988 |
| GOTERM_BP_FAT | GO:0009664~plant-type cell wall organization | 0.01127 |
| GOTERM_BP_FAT | GO:0015893~drug transport | 0.01318 |
| GOTERM_BP_FAT | GO:0016246~RNA interference | 0.014998 |
| GOTERM_BP_FAT | GO:0042493~response to drug | 0.015334 |
| GOTERM_BP_FAT | GO:0045229~external encapsulating structure organization | 0.015657 |
| GOTERM_BP_FAT | GO:0030245~cellulose catabolic process | 0.02359 |
| GOTERM_BP_FAT | GO:0006350~transcription | 0.026596 |
| GOTERM_BP_FAT | GO:0030005~cellular di-, tri-valent inorganic cation homeostasis | 0.028021 |
| GOTERM_BP_FAT | GO:0055066~di-, tri-valent inorganic cation homeostasis | 0.031283 |
| GOTERM_BP_FAT | GO:0040029~regulation of gene expression, epigenetic | 0.036647 |
| GOTERM_BP_FAT | GO:0010267~RNA interference, production of ta-siRNAs | 0.036881 |
| GOTERM_BP_FAT | GO:0044092~negative regulation of molecular function | 0.052771 |
| GOTERM_BP_FAT | GO:0016458~gene silencing | 0.065879 |
| GOTERM_BP_FAT | GO:0043086~negative regulation of catalytic activity | 0.066169 |
| GOTERM_BP_FAT | GO:0031047~gene silencing by RNA | 0.069337 |
| GOTERM_BP_FAT | GO:0010098~suspensor development | 0.092697 |
| GOTERM_CC_FAT | GO:0012505~endomembrane system | 1.15E-27 |
| GOTERM_CC_FAT | GO:0031224~intrinsic to membrane | 0.044814 |
| GOTERM_CC_FAT | GO:0005667~transcription factor complex | 0.073103 |
| GOTERM_MF_FAT | GO:0004674~protein serine/threonine kinase activity | 1.90E-09 |
| GOTERM_MF_FAT | GO:0004672~protein kinase activity | 7.54E-09 |
| GOTERM_MF_FAT | GO:0005524~ATP binding | 1.85E-08 |
| GOTERM_MF_FAT | GO:0032559~adenyl ribonucleotide binding | 3.53E-08 |
| GOTERM_MF_FAT | GO:0001882~nucleoside binding | 1.10E-07 |
| GOTERM_MF_FAT | GO:0032555~purine ribonucleotide binding | 1.41E-07 |
| GOTERM_MF_FAT | GO:0032553~ribonucleotide binding | 1.41E-07 |
| GOTERM_MF_FAT | GO:0030554~adenyl nucleotide binding | 1.61E-07 |
| GOTERM_MF_FAT | GO:0001883~purine nucleoside binding | 1.61E-07 |
| GOTERM_MF_FAT | GO:0017076~purine nucleotide binding | 6.77E-07 |
| GOTERM_MF_FAT | GO:0005529~sugar binding | 3.41E-05 |
| GOTERM_MF_FAT | GO:0000166~nucleotide binding | 1.75E-04 |
| GOTERM_MF_FAT | GO:0004713~protein tyrosine kinase activity | 1.96E-04 |
| GOTERM_MF_FAT | GO:0030246~carbohydrate binding | 2.14E-04 |
| GOTERM_MF_FAT | GO:0003700~transcription factor activity | 6.41E-04 |
| GOTERM_MF_FAT | GO:0005234~extracellular-glutamate-gated ion channel activity | 8.38E-04 |
| GOTERM_MF_FAT | GO:0004970~ionotropic glutamate receptor activity | 8.38E-04 |
| GOTERM_MF_FAT | GO:0008066~glutamate receptor activity | 8.38E-04 |
| GOTERM_MF_FAT | GO:0005230~extracellular ligand-gated ion channel activity | 8.38E-04 |
| GOTERM_MF_FAT | GO:0015238~drug transporter activity | 0.001627 |
| GOTERM_MF_FAT | GO:0003677~DNA binding | 0.001791 |
| GOTERM_MF_FAT | GO:0030528~transcription regulator activity | 0.00225 |
| GOTERM_MF_FAT | GO:0005199~structural constituent of cell wall | 0.002289 |
| GOTERM_MF_FAT | GO:0005217~intracellular ligand-gated ion channel activity | 0.002429 |
| GOTERM_MF_FAT | GO:0016820~hydrolase activity, acting on acid anhydrides, catalyzing transmembrane movement of substances | 0.002926 |
| GOTERM_MF_FAT | GO:0043492~ATPase activity, coupled to movement of substances | 0.003513 |
| GOTERM_MF_FAT | GO:0042626~ATPase activity, coupled to transmembrane movement of substances | 0.003513 |
| GOTERM_MF_FAT | GO:0022834~ligand-gated channel activity | 0.003878 |
| GOTERM_MF_FAT | GO:0015276~ligand-gated ion channel activity | 0.003878 |
| GOTERM_MF_FAT | GO:0022836~gated channel activity | 0.018617 |
| GOTERM_MF_FAT | GO:0015405~P-P-bond-hydrolysis-driven transmembrane transporter activity | 0.019398 |
| GOTERM_MF_FAT | GO:0015399~primary active transmembrane transporter activity | 0.020964 |
| GOTERM_MF_FAT | GO:0042623~ATPase activity, coupled | 0.02386 |
| GOTERM_MF_FAT | GO:0005216~ion channel activity | 0.029142 |
| GOTERM_MF_FAT | GO:0015297~antiporter activity | 0.032544 |
| GOTERM_MF_FAT | GO:0004185~serine-type carboxypeptidase activity | 0.04819 |
| GOTERM_MF_FAT | GO:0070008~serine-type exopeptidase activity | 0.04819 |
| GOTERM_MF_FAT | GO:0004180~carboxypeptidase activity | 0.053266 |
| GOTERM_MF_FAT | GO:0008236~serine-type peptidase activity | 0.056928 |
| GOTERM_MF_FAT | GO:0017171~serine hydrolase activity | 0.056928 |
| GOTERM_MF_FAT | GO:0003774~motor activity | 0.059217 |
| GOTERM_MF_FAT | GO:0004806~triacylglycerol lIPACse activity | 0.065818 |
| GOTERM_MF_FAT | GO:0015271~outward rectifier potassium channel activity | 0.097284 |
| GOTERM_MF_FAT | GO:0016917~GABA receptor activity | 0.097284 |
| GOTERM_MF_FAT | GO:0004965~GABA-B receptor activity | 0.097284 |
| GOTERM_MF_FAT | GO:0008810~cellulase activity | 0.097433 |

Table S2. Functional classes over-represented in the set of genes associated with A-IPACs

| Category | Term | PValue |
| --- | --- | --- |
| GOTERM_BP_FAT | GO:0009628~response to abiotic stimulus | 1.72E-22 |
| GOTERM_BP_FAT | GO:0009409~response to cold | 2.69E-15 |
| GOTERM_BP_FAT | GO:0009266~response to temperature stimulus | 5.13E-13 |
| GOTERM_BP_FAT | GO:0006970~response to osmotic stress | 2.36E-11 |
| GOTERM_BP_FAT | GO:0009651~response to salt stress | 1.23E-09 |
| GOTERM_BP_FAT | GO:0010033~response to organic substance | 1.72E-09 |
| GOTERM_BP_FAT | GO:0009611~response to wounding | 5.63E-08 |
| GOTERM_BP_FAT | GO:0046686~response to cadmium ion | 1.16E-07 |
| GOTERM_BP_FAT | GO:0016051~carbohydrate biosynthetic process | 1.43E-07 |
| GOTERM_BP_FAT | GO:0010038~response to metal ion | 3.77E-07 |
| GOTERM_BP_FAT | GO:0010035~response to inorganic substance | 8.03E-07 |
| GOTERM_BP_FAT | GO:0009416~response to light stimulus | 1.67E-06 |
| GOTERM_BP_FAT | GO:0055114~oxidation reduction | 3.11E-06 |
| GOTERM_BP_FAT | GO:0009314~response to radiation | 7.47E-06 |
| GOTERM_BP_FAT | GO:0009719~response to endogenous stimulus | 1.21E-05 |
| GOTERM_BP_FAT | GO:0006631~fatty acid metabolic process | 1.85E-05 |
| GOTERM_BP_FAT | GO:0009617~response to bacterium | 1.99E-05 |
| GOTERM_BP_FAT | GO:0006091~generation of precursor metabolites and energy | 2.01E-05 |
| GOTERM_BP_FAT | GO:0015979~photosynthesis | 2.21E-05 |
| GOTERM_BP_FAT | GO:0006633~fatty acid biosynthetic process | 5.87E-05 |
| GOTERM_BP_FAT | GO:0009725~response to hormone stimulus | 1.05E-04 |
| GOTERM_BP_FAT | GO:0042742~defense response to bacterium | 1.63E-04 |
| GOTERM_BP_FAT | GO:0016143~S-glycoside metabolic process | 1.90E-04 |
| GOTERM_BP_FAT | GO:0019757~glycosinolate metabolic process | 1.90E-04 |
| GOTERM_BP_FAT | GO:0019760~glucosinolate metabolic process | 1.90E-04 |
| GOTERM_BP_FAT | GO:0040007~growth | 1.94E-04 |
| GOTERM_BP_FAT | GO:0016137~glycoside metabolic process | 2.01E-04 |
| GOTERM_BP_FAT | GO:0006090~pyruvate metabolic process | 2.18E-04 |
| GOTERM_BP_FAT | GO:0009743~response to carbohydrate stimulus | 2.35E-04 |
| GOTERM_BP_FAT | GO:0016053~organic acid biosynthetic process | 2.51E-04 |
| GOTERM_BP_FAT | GO:0046394~carboxylic acid biosynthetic process | 2.51E-04 |
| GOTERM_BP_FAT | GO:0009639~response to red or far red light | 5.36E-04 |
| GOTERM_BP_FAT | GO:0006096~glycolysis | 5.77E-04 |
| GOTERM_BP_FAT | GO:0034637~cellular carbohydrate biosynthetic process | 5.92E-04 |
| GOTERM_BP_FAT | GO:0048364~root development | 6.52E-04 |
| GOTERM_BP_FAT | GO:0022622~root system development | 6.52E-04 |
| GOTERM_BP_FAT | GO:0005996~monosaccharide metabolic process | 7.46E-04 |
| GOTERM_BP_FAT | GO:0006081~cellular aldehyde metabolic process | 7.59E-04 |
| GOTERM_BP_FAT | GO:0016052~carbohydrate catabolic process | 7.92E-04 |
| GOTERM_BP_FAT | GO:0009753~response to jasmonic acid stimulus | 8.60E-04 |
| GOTERM_BP_FAT | GO:0044275~cellular carbohydrate catabolic process | 9.63E-04 |
| GOTERM_BP_FAT | GO:0006006~glucose metabolic process | 0.001007 |
| GOTERM_BP_FAT | GO:0008361~regulation of cell size | 0.001096 |
| GOTERM_BP_FAT | GO:0042445~hormone metabolic process | 0.001138 |
| GOTERM_BP_FAT | GO:0016049~cell growth | 0.001156 |
| GOTERM_BP_FAT | GO:0019318~hexose metabolic process | 0.001219 |
| GOTERM_BP_FAT | GO:0009765~photosynthesis, light harvesting | 0.001246 |
| GOTERM_BP_FAT | GO:0009755~hormone-mediated signaling | 0.00141 |
| GOTERM_BP_FAT | GO:0032870~cellular response to hormone stimulus | 0.00141 |
| GOTERM_BP_FAT | GO:0010817~regulation of hormone levels | 0.001509 |
| GOTERM_BP_FAT | GO:0019684~photosynthesis, light reaction | 0.001601 |
| GOTERM_BP_FAT | GO:0008610~lipid biosynthetic process | 0.001709 |
| GOTERM_BP_FAT | GO:0009737~response to abscisic acid stimulus | 0.002112 |
| GOTERM_BP_FAT | GO:0046164~alcohol catabolic process | 0.002194 |
| GOTERM_BP_FAT | GO:0009749~response to glucose stimulus | 0.002386 |
| GOTERM_BP_FAT | GO:0006007~glucose catabolic process | 0.002419 |
| GOTERM_BP_FAT | GO:0046365~monosaccharide catabolic process | 0.002762 |
| GOTERM_BP_FAT | GO:0019320~hexose catabolic process | 0.002762 |
| GOTERM_BP_FAT | GO:0042446~hormone biosynthetic process | 0.002854 |
| GOTERM_BP_FAT | GO:0032535~regulation of cellular component size | 0.003172 |
| GOTERM_BP_FAT | GO:0008202~steroid metabolic process | 0.003186 |
| GOTERM_BP_FAT | GO:0009746~response to hexose stimulus | 0.003247 |
| GOTERM_BP_FAT | GO:0034284~response to monosaccharide stimulus | 0.003247 |
| GOTERM_BP_FAT | GO:0009694~jasmonic acid metabolic process | 0.003247 |
| GOTERM_BP_FAT | GO:0016138~glycoside biosynthetic process | 0.003466 |
| GOTERM_BP_FAT | GO:0006979~response to oxidative stress | 0.004039 |
| GOTERM_BP_FAT | GO:0019748~secondary metabolic process | 0.00432 |
| GOTERM_BP_FAT | GO:0052543~callose deposition in cell wall | 0.004436 |
| GOTERM_BP_FAT | GO:0042430~indole and derivative metabolic process | 0.004451 |
| GOTERM_BP_FAT | GO:0042434~indole derivative metabolic process | 0.004451 |
| GOTERM_BP_FAT | GO:0042435~indole derivative biosynthetic process | 0.004561 |
| GOTERM_BP_FAT | GO:0009646~response to absence of light | 0.004798 |
| GOTERM_BP_FAT | GO:0019253~reductive pentose-phosphate cycle | 0.004798 |
| GOTERM_BP_FAT | GO:0009695~jasmonic acid biosynthetic process | 0.005274 |
| GOTERM_BP_FAT | GO:0010015~root morphogenesis | 0.005709 |
| GOTERM_BP_FAT | GO:0007242~intracellular signaling cascade | 0.006195 |
| GOTERM_BP_FAT | GO:0052386~cell wall thickening | 0.00627 |
| GOTERM_BP_FAT | GO:0009741~response to brassinosteroid stimulus | 0.006692 |
| GOTERM_BP_FAT | GO:0010218~response to far red light | 0.006692 |
| GOTERM_BP_FAT | GO:0009850~auxin metabolic process | 0.00706 |
| GOTERM_BP_FAT | GO:0019685~photosynthesis, dark reaction | 0.007155 |
| GOTERM_BP_FAT | GO:0031407~oxylipin metabolic process | 0.007304 |
| GOTERM_BP_FAT | GO:0019758~glycosinolate biosynthetic process | 0.007342 |
| GOTERM_BP_FAT | GO:0019761~glucosinolate biosynthetic process | 0.007342 |
| GOTERM_BP_FAT | GO:0016144~S-glycoside biosynthetic process | 0.007342 |
| GOTERM_BP_FAT | GO:0010200~response to chitin | 0.007834 |
| GOTERM_BP_FAT | GO:0010114~response to red light | 0.008148 |
| GOTERM_BP_FAT | GO:0006800~oxygen and reactive oxygen species metabolic process | 0.00826 |
| GOTERM_BP_FAT | GO:0015977~carbon utilization by fixation of carbon dioxide | 0.008616 |
| GOTERM_BP_FAT | GO:0052545~callose localization | 0.008616 |
| GOTERM_BP_FAT | GO:0009738~abscisic acid mediated signaling | 0.00926 |
| GOTERM_BP_FAT | GO:0031408~oxylipin biosynthetic process | 0.009341 |
| GOTERM_BP_FAT | GO:0051186~cofactor metabolic process | 0.009342 |
| GOTERM_BP_FAT | GO:0048589~developmental growth | 0.009569 |
| GOTERM_BP_FAT | GO:0052544~callose deposition in cell wall during defense response | 0.010247 |
| GOTERM_BP_FAT | GO:0052482~cell wall thickening during defense response | 0.010247 |
| GOTERM_BP_FAT | GO:0060560~developmental growth involved in morphogenesis | 0.011189 |
| GOTERM_BP_FAT | GO:0009826~unidimensional cell growth | 0.011189 |
| GOTERM_BP_FAT | GO:0002213~defense response to insect | 0.011483 |
| GOTERM_BP_FAT | GO:0009636~response to toxin | 0.011546 |
| GOTERM_BP_FAT | GO:0009851~auxin biosynthetic process | 0.011546 |
| GOTERM_BP_FAT | GO:0033037~polysaccharide localization | 0.011546 |
| GOTERM_BP_FAT | GO:0009739~response to gibberellin stimulus | 0.012405 |
| GOTERM_BP_FAT | GO:0009820~alkaloid metabolic process | 0.013036 |
| GOTERM_BP_FAT | GO:0006790~sulfur metabolic process | 0.013333 |
| GOTERM_BP_FAT | GO:0046395~carboxylic acid catabolic process | 0.013762 |
| GOTERM_BP_FAT | GO:0016054~organic acid catabolic process | 0.013762 |
| GOTERM_BP_FAT | GO:0034599~cellular response to oxidative stress | 0.015102 |
| GOTERM_BP_FAT | GO:0033554~cellular response to stress | 0.015663 |
| GOTERM_BP_FAT | GO:0022900~electron transport chain | 0.015672 |
| GOTERM_BP_FAT | GO:0002238~response to molecule of fungal origin | 0.016323 |
| GOTERM_BP_FAT | GO:0019759~glycosinolate catabolic process | 0.016865 |
| GOTERM_BP_FAT | GO:0019762~glucosinolate catabolic process | 0.016865 |
| GOTERM_BP_FAT | GO:0016145~S-glycoside catabolic process | 0.016865 |
| GOTERM_BP_FAT | GO:0008654~phospholipid biosynthetic process | 0.01762 |
| GOTERM_BP_FAT | GO:0048585~negative regulation of response to stimulus | 0.017624 |
| GOTERM_BP_FAT | GO:0009642~response to light intensity | 0.017691 |
| GOTERM_BP_FAT | GO:0044273~sulfur compound catabolic process | 0.019052 |
| GOTERM_BP_FAT | GO:0052542~callose deposition during defense response | 0.019052 |
| GOTERM_BP_FAT | GO:0009414~response to water deprivation | 0.019786 |
| GOTERM_BP_FAT | GO:0006073~cellular glucan metabolic process | 0.020069 |
| GOTERM_BP_FAT | GO:0015994~chlorophyll metabolic process | 0.021137 |
| GOTERM_BP_FAT | GO:0015995~chlorophyll biosynthetic process | 0.021596 |
| GOTERM_BP_FAT | GO:0044042~glucan metabolic process | 0.021991 |
| GOTERM_BP_FAT | GO:0031668~cellular response to extracellular stimulus | 0.022254 |
| GOTERM_BP_FAT | GO:0009415~response to water | 0.023525 |
| GOTERM_BP_FAT | GO:0009625~response to insect | 0.023569 |
| GOTERM_BP_FAT | GO:0016139~glycoside catabolic process | 0.023569 |
| GOTERM_BP_FAT | GO:0005976~polysaccharide metabolic process | 0.024043 |
| GOTERM_BP_FAT | GO:0010053~root epidermal cell differentiation | 0.02472 |
| GOTERM_BP_FAT | GO:0034050~host programmed cell death induced by symbiont | 0.02472 |
| GOTERM_BP_FAT | GO:0034614~cellular response to reactive oxygen species | 0.026247 |
| GOTERM_BP_FAT | GO:0009684~indoleacetic acid biosynthetic process | 0.02801 |
| GOTERM_BP_FAT | GO:0000902~cell morphogenesis | 0.028441 |
| GOTERM_BP_FAT | GO:0009310~amine catabolic process | 0.028723 |
| GOTERM_BP_FAT | GO:0010193~response to ozone | 0.0305 |
| GOTERM_BP_FAT | GO:0019362~pyridine nucleotide metabolic process | 0.033171 |
| GOTERM_BP_FAT | GO:0032989~cellular component morphogenesis | 0.033218 |
| GOTERM_BP_FAT | GO:0009064~glutamine family amino acid metabolic process | 0.033378 |
| GOTERM_BP_FAT | GO:0034754~cellular hormone metabolic process | 0.034117 |
| GOTERM_BP_FAT | GO:0043623~cellular protein complex assembly | 0.035254 |
| GOTERM_BP_FAT | GO:0048764~trichoblast maturation | 0.036342 |
| GOTERM_BP_FAT | GO:0048469~cell maturation | 0.036342 |
| GOTERM_BP_FAT | GO:0016036~cellular response to phosphate starvation | 0.036342 |
| GOTERM_BP_FAT | GO:0048765~root hair cell differentiation | 0.036342 |
| GOTERM_BP_FAT | GO:0048545~response to steroid hormone stimulus | 0.037347 |
| GOTERM_BP_FAT | GO:0009225~nucleotide-sugar metabolic process | 0.037347 |
| GOTERM_BP_FAT | GO:0009742~brassinosteroid mediated signaling | 0.037347 |
| GOTERM_BP_FAT | GO:0043401~steroid hormone mediated signaling | 0.037347 |
| GOTERM_BP_FAT | GO:0006778~porphyrin metabolic process | 0.03771 |
| GOTERM_BP_FAT | GO:0009637~response to blue light | 0.038082 |
| GOTERM_BP_FAT | GO:0009683~indoleacetic acid metabolic process | 0.039724 |
| GOTERM_BP_FAT | GO:0009991~response to extracellular stimulus | 0.040133 |
| GOTERM_BP_FAT | GO:0010476~gibberellin-mediated signaling | 0.040645 |
| GOTERM_BP_FAT | GO:0009740~gibberellic acid mediated signaling | 0.040645 |
| GOTERM_BP_FAT | GO:0033013~tetrapyrrole metabolic process | 0.042426 |
| GOTERM_BP_FAT | GO:0048528~post-embryonic root development | 0.043129 |
| GOTERM_BP_FAT | GO:0006733~oxidoreduction coenzyme metabolic process | 0.043129 |
| GOTERM_BP_FAT | GO:0009063~cellular amino acid catabolic process | 0.04334 |
| GOTERM_BP_FAT | GO:0006694~steroid biosynthetic process | 0.04334 |
| GOTERM_BP_FAT | GO:0046838~phosphorylated carbohydrate dephosphorylation | 0.04348 |
| GOTERM_BP_FAT | GO:0046855~inositol phosphate dephosphorylation | 0.04348 |
| GOTERM_BP_FAT | GO:0033014~tetrapyrrole biosynthetic process | 0.043482 |
| GOTERM_BP_FAT | GO:0009250~glucan biosynthetic process | 0.044814 |
| GOTERM_BP_FAT | GO:0031669~cellular response to nutrient levels | 0.046248 |
| GOTERM_BP_FAT | GO:0006094~gluconeogenesis | 0.047043 |
| GOTERM_BP_FAT | GO:0042343~indole glucosinolate metabolic process | 0.047043 |
| GOTERM_BP_FAT | GO:0015031~protein transport | 0.04903 |
| GOTERM_BP_FAT | GO:0045184~establishment of protein localization | 0.04903 |
| GOTERM_BP_FAT | GO:0009069~serine family amino acid metabolic process | 0.049383 |
| GOTERM_BP_FAT | GO:0048527~lateral root development | 0.049383 |
| GOTERM_BP_FAT | GO:0019932~second-messenger-mediated signaling | 0.049483 |
| GOTERM_BP_FAT | GO:0009626~plant-type hypersensitive response | 0.049684 |
| GOTERM_BP_FAT | GO:0009814~defense response, incompatible interaction | 0.052609 |
| GOTERM_BP_FAT | GO:0045087~innate immune response | 0.054454 |
| GOTERM_BP_FAT | GO:0044271~nitrogen compound biosynthetic process | 0.055434 |
| GOTERM_BP_FAT | GO:0009640~photomorphogenesis | 0.056619 |
| GOTERM_BP_FAT | GO:0010054~trichoblast differentiation | 0.056809 |
| GOTERM_BP_FAT | GO:0008380~RNA splicing | 0.060926 |
| GOTERM_BP_FAT | GO:0022613~ribonucleoprotein complex biogenesis | 0.060998 |
| GOTERM_BP_FAT | GO:0042254~ribosome biogenesis | 0.061171 |
| GOTERM_BP_FAT | GO:0051188~cofactor biosynthetic process | 0.063345 |
| GOTERM_BP_FAT | GO:0015672~monovalent inorganic cation transport | 0.063345 |
| GOTERM_BP_FAT | GO:0006779~porphyrin biosynthetic process | 0.064158 |
| GOTERM_BP_FAT | GO:0016125~sterol metabolic process | 0.06499 |
| GOTERM_BP_FAT | GO:0006955~immune response | 0.065177 |
| GOTERM_BP_FAT | GO:0043094~cellular metabolic compound salvage | 0.065393 |
| GOTERM_BP_FAT | GO:0009269~response to desiccation | 0.066017 |
| GOTERM_BP_FAT | GO:0019319~hexose biosynthetic process | 0.066017 |
| GOTERM_BP_FAT | GO:0010100~negative regulation of photomorphogenesis | 0.068379 |
| GOTERM_BP_FAT | GO:0006995~cellular response to nitrogen starvation | 0.068379 |
| GOTERM_BP_FAT | GO:0000578~embryonic axis specification | 0.068379 |
| GOTERM_BP_FAT | GO:0031399~regulation of protein modification process | 0.068379 |
| GOTERM_BP_FAT | GO:0051248~negative regulation of protein metabolic process | 0.068379 |
| GOTERM_BP_FAT | GO:0032269~negative regulation of cellular protein metabolic process | 0.068379 |
| GOTERM_BP_FAT | GO:0008104~protein localization | 0.069423 |
| GOTERM_BP_FAT | GO:0043562~cellular response to nitrogen levels | 0.070226 |
| GOTERM_BP_FAT | GO:0006813~potassium ion transport | 0.070246 |
| GOTERM_BP_FAT | GO:0009913~epidermal cell differentiation | 0.070883 |
| GOTERM_BP_FAT | GO:0042594~response to starvation | 0.072211 |
| GOTERM_BP_FAT | GO:0021700~developmental maturation | 0.072313 |
| GOTERM_BP_FAT | GO:0009853~photorespiration | 0.073911 |
| GOTERM_BP_FAT | GO:0070727~cellular macromolecule localization | 0.073982 |
| GOTERM_BP_FAT | GO:0044272~sulfur compound biosynthetic process | 0.074842 |
| GOTERM_BP_FAT | GO:0006812~cation transport | 0.075201 |
| GOTERM_BP_FAT | GO:0009267~cellular response to starvation | 0.075292 |
| GOTERM_BP_FAT | GO:0031667~response to nutrient levels | 0.078849 |
| GOTERM_BP_FAT | GO:0042545~cell wall modification | 0.079178 |
| GOTERM_BP_FAT | GO:0009759~indole glucosinolate biosynthetic process | 0.079757 |
| GOTERM_BP_FAT | GO:0080027~response to herbivore | 0.079757 |
| GOTERM_BP_FAT | GO:0048830~adventitious root development | 0.079757 |
| GOTERM_BP_FAT | GO:0009939~positive regulation of gibberellic acid mediated signaling | 0.079757 |
| GOTERM_BP_FAT | GO:0046364~monosaccharide biosynthetic process | 0.080683 |
| GOTERM_BP_FAT | GO:0008544~epidermis development | 0.081889 |
| GOTERM_BP_FAT | GO:0007398~ectoderm development | 0.081889 |
| GOTERM_BP_FAT | GO:0009751~response to salicylic acid stimulus | 0.085344 |
| GOTERM_BP_FAT | GO:0034285~response to disaccharide stimulus | 0.085346 |
| GOTERM_BP_FAT | GO:0006952~defense response | 0.08863 |
| GOTERM_BP_FAT | GO:0019722~calcium-mediated signaling | 0.088889 |
| GOTERM_BP_FAT | GO:0006801~superoxide metabolic process | 0.088889 |
| GOTERM_BP_FAT | GO:0010099~regulation of photomorphogenesis | 0.088889 |
| GOTERM_BP_FAT | GO:0045454~cell redox homeostasis | 0.090366 |
| GOTERM_BP_FAT | GO:0046496~nicotinamide nucleotide metabolic process | 0.093866 |
| GOTERM_BP_FAT | GO:0006769~nicotinamide metabolic process | 0.093866 |
| GOTERM_BP_FAT | GO:0009734~auxin mediated signaling pathway | 0.09397 |
| GOTERM_BP_FAT | GO:0006811~ion transport | 0.096769 |
| GOTERM_BP_FAT | GO:0009312~oligosaccharide biosynthetic process | 0.096773 |
| GOTERM_BP_FAT | GO:0006739~NADP metabolic process | 0.096773 |
| GOTERM_BP_FAT | GO:0009631~cold acclimation | 0.096927 |
| GOTERM_BP_FAT | GO:0009817~defense response to fungus, incompatible interaction | 0.099311 |
| GOTERM_BP_FAT | GO:0048767~root hair elongation | 0.099311 |
| GOTERM_BP_FAT | GO:0006732~coenzyme metabolic process | 0.099784 |
| GOTERM_CC_FAT | GO:0044434~chloroplast part | 7.21E-22 |
| GOTERM_CC_FAT | GO:0044435~plastid part | 4.30E-21 |
| GOTERM_CC_FAT | GO:0005886~plasma membrane | 9.25E-18 |
| GOTERM_CC_FAT | GO:0009941~chloroplast envelope | 3.65E-17 |
| GOTERM_CC_FAT | GO:0009526~plastid envelope | 2.10E-16 |
| GOTERM_CC_FAT | GO:0009579~thylakoid | 2.80E-14 |
| GOTERM_CC_FAT | GO:0009570~chloroplast stroma | 1.43E-13 |
| GOTERM_CC_FAT | GO:0009532~plastid stroma | 3.79E-13 |
| GOTERM_CC_FAT | GO:0009534~chloroplast thylakoid | 4.16E-13 |
| GOTERM_CC_FAT | GO:0031976~plastid thylakoid | 4.16E-13 |
| GOTERM_CC_FAT | GO:0031984~organelle subcompartment | 6.04E-13 |
| GOTERM_CC_FAT | GO:0031090~organelle membrane | 7.50E-13 |
| GOTERM_CC_FAT | GO:0042651~thylakoid membrane | 9.53E-13 |
| GOTERM_CC_FAT | GO:0009535~chloroplast thylakoid membrane | 2.76E-12 |
| GOTERM_CC_FAT | GO:0055035~plastid thylakoid membrane | 2.76E-12 |
| GOTERM_CC_FAT | GO:0044436~thylakoid part | 3.82E-12 |
| GOTERM_CC_FAT | GO:0005773~vacuole | 2.85E-11 |
| GOTERM_CC_FAT | GO:0031975~envelope | 3.19E-11 |
| GOTERM_CC_FAT | GO:0034357~photosynthetic membrane | 3.69E-11 |
| GOTERM_CC_FAT | GO:0030312~external encapsulating structure | 6.08E-11 |
| GOTERM_CC_FAT | GO:0031967~organelle envelope | 6.11E-11 |
| GOTERM_CC_FAT | GO:0005618~cell wall | 1.69E-10 |
| GOTERM_CC_FAT | GO:0005829~cytosol | 3.88E-10 |
| GOTERM_CC_FAT | GO:0022626~cytosolic ribosome | 8.98E-10 |
| GOTERM_CC_FAT | GO:0010287~plastoglobule | 1.18E-09 |
| GOTERM_CC_FAT | GO:0009507~chloroplast | 3.01E-09 |
| GOTERM_CC_FAT | GO:0009536~plastid | 9.57E-09 |
| GOTERM_CC_FAT | GO:0048046~apoplast | 7.88E-08 |
| GOTERM_CC_FAT | GO:0005840~ribosome | 1.02E-06 |
| GOTERM_CC_FAT | GO:0044445~cytosolic part | 2.34E-06 |
| GOTERM_CC_FAT | GO:0009505~plant-type cell wall | 1.78E-05 |
| GOTERM_CC_FAT | GO:0033279~ribosomal subunit | 1.26E-04 |
| GOTERM_CC_FAT | GO:0010319~stromule | 1.34E-04 |
| GOTERM_CC_FAT | GO:0009522~photosystem I | 1.39E-04 |
| GOTERM_CC_FAT | GO:0030529~ribonucleoprotein complex | 2.15E-04 |
| GOTERM_CC_FAT | GO:0009521~photosystem | 2.32E-04 |
| GOTERM_CC_FAT | GO:0022627~cytosolic small ribosomal subunit | 5.71E-04 |
| GOTERM_CC_FAT | GO:0031974~membrane-enclosed lumen | 0.001178 |
| GOTERM_CC_FAT | GO:0070013~intracellular organelle lumen | 0.001588 |
| GOTERM_CC_FAT | GO:0043233~organelle lumen | 0.001588 |
| GOTERM_CC_FAT | GO:0005783~endoplasmic reticulum | 0.002442 |
| GOTERM_CC_FAT | GO:0031224~intrinsic to membrane | 0.002457 |
| GOTERM_CC_FAT | GO:0005774~vacuolar membrane | 0.00265 |
| GOTERM_CC_FAT | GO:0015935~small ribosomal subunit | 0.003295 |
| GOTERM_CC_FAT | GO:0000325~plant-type vacuole | 0.003601 |
| GOTERM_CC_FAT | GO:0044437~vacuolar part | 0.004183 |
| GOTERM_CC_FAT | GO:0009528~plastid inner membrane | 0.00693 |
| GOTERM_CC_FAT | GO:0030076~light-harvesting complex | 0.007137 |
| GOTERM_CC_FAT | GO:0005730~nucleolus | 0.007494 |
| GOTERM_CC_FAT | GO:0048492~ribulose bisphosphate carboxylase complex | 0.007956 |
| GOTERM_CC_FAT | GO:0009573~chloroplast ribulose bisphosphate carboxylase complex | 0.007956 |
| GOTERM_CC_FAT | GO:0016021~integral to membrane | 0.008344 |
| GOTERM_CC_FAT | GO:0022625~cytosolic large ribosomal subunit | 0.010614 |
| GOTERM_CC_FAT | GO:0008540~proteasome regulatory particle, base subcomplex | 0.013345 |
| GOTERM_CC_FAT | GO:0005838~proteasome regulatory particle | 0.014534 |
| GOTERM_CC_FAT | GO:0022624~proteasome accessory complex | 0.014534 |
| GOTERM_CC_FAT | GO:0043232~intracellular non-membrane-bounded organelle | 0.016298 |
| GOTERM_CC_FAT | GO:0043228~non-membrane-bounded organelle | 0.016298 |
| GOTERM_CC_FAT | GO:0009706~chloroplast inner membrane | 0.017151 |
| GOTERM_CC_FAT | GO:0031225~anchored to membrane | 0.01809 |
| GOTERM_CC_FAT | GO:0042170~plastid membrane | 0.01913 |
| GOTERM_CC_FAT | GO:0042579~microbody | 0.020349 |
| GOTERM_CC_FAT | GO:0005777~peroxisome | 0.020349 |
| GOTERM_CC_FAT | GO:0031977~thylakoid lumen | 0.021383 |
| GOTERM_CC_FAT | GO:0030095~chloroplast photosystem II | 0.022296 |
| GOTERM_CC_FAT | GO:0015934~large ribosomal subunit | 0.024494 |
| GOTERM_CC_FAT | GO:0009523~photosystem II | 0.032291 |
| GOTERM_CC_FAT | GO:0031969~chloroplast membrane | 0.037786 |
| GOTERM_CC_FAT | GO:0046658~anchored to plasma membrane | 0.042479 |
| GOTERM_CC_FAT | GO:0005794~Golgi apparatus | 0.044966 |
| GOTERM_CC_FAT | GO:0005835~fatty acid synthase complex | 0.046422 |
| GOTERM_CC_FAT | GO:0031980~mitochondrial lumen | 0.046968 |
| GOTERM_CC_FAT | GO:0005759~mitochondrial matrix | 0.046968 |
| GOTERM_CC_FAT | GO:0031981~nuclear lumen | 0.0647 |
| GOTERM_CC_FAT | GO:0030054~cell junction | 0.068987 |
| GOTERM_CC_FAT | GO:0009543~chloroplast thylakoid lumen | 0.069795 |
| GOTERM_CC_FAT | GO:0031978~plastid thylakoid lumen | 0.069795 |
| GOTERM_CC_FAT | GO:0005911~cell-cell junction | 0.074529 |
| GOTERM_CC_FAT | GO:0000502~proteasome complex | 0.083432 |
| GOTERM_MF_FAT | GO:0005198~structural molecule activity | 1.05E-05 |
| GOTERM_MF_FAT | GO:0004028~3-chloroallyl aldehyde dehydrogenase activity | 9.93E-05 |
| GOTERM_MF_FAT | GO:0003735~structural constituent of ribosome | 1.99E-04 |
| GOTERM_MF_FAT | GO:0008266~poly(U) RNA binding | 3.83E-04 |
| GOTERM_MF_FAT | GO:0008187~poly-pyrimidine tract binding | 3.83E-04 |
| GOTERM_MF_FAT | GO:0016831~carboxy-lyase activity | 0.001144 |
| GOTERM_MF_FAT | GO:0016620~oxidoreductase activity, acting on the aldehyde or oxo group of donors, NAD or NADP as acceptor | 0.001578 |
| GOTERM_MF_FAT | GO:0048037~cofactor binding | 0.001735 |
| GOTERM_MF_FAT | GO:0003727~single-stranded RNA binding | 0.002182 |
| GOTERM_MF_FAT | GO:0004332~fructose-bisphosphate aldolase activity | 0.002675 |
| GOTERM_MF_FAT | GO:0004177~aminopeptidase activity | 0.002956 |
| GOTERM_MF_FAT | GO:0009055~electron carrier activity | 0.005045 |
| GOTERM_MF_FAT | GO:0016854~racemase and epimerase activity | 0.006199 |
| GOTERM_MF_FAT | GO:0008235~metalloexopeptidase activity | 0.007551 |
| GOTERM_MF_FAT | GO:0016168~chlorophyll binding | 0.007885 |
| GOTERM_MF_FAT | GO:0046906~tetrapyrrole binding | 0.008103 |
| GOTERM_MF_FAT | GO:0008943~glyceraldehyde-3-phosphate dehydrogenase activity | 0.011937 |
| GOTERM_MF_FAT | GO:0004029~aldehyde dehydrogenase (NAD) activity | 0.011937 |
| GOTERM_MF_FAT | GO:0030145~manganese ion binding | 0.015585 |
| GOTERM_MF_FAT | GO:0016857~racemase and epimerase activity, acting on carbohydrates and derivatives | 0.019032 |
| GOTERM_MF_FAT | GO:0043176~amine binding | 0.019355 |
| GOTERM_MF_FAT | GO:0019904~protein domain specific binding | 0.020032 |
| GOTERM_MF_FAT | GO:0046527~glucosyltransferase activity | 0.022981 |
| GOTERM_MF_FAT | GO:0045309~protein phosphorylated amino acid binding | 0.024614 |
| GOTERM_MF_FAT | GO:0051219~phosphoprotein binding | 0.024614 |
| GOTERM_MF_FAT | GO:0005507~copper ion binding | 0.027614 |
| GOTERM_MF_FAT | GO:0050662~coenzyme binding | 0.028984 |
| GOTERM_MF_FAT | GO:0070569~uridylyltransferase activity | 0.030494 |
| GOTERM_MF_FAT | GO:0047262~polygalacturonate 4-alpha-galacturonosyltransferase activity | 0.032164 |
| GOTERM_MF_FAT | GO:0004312~fatty-acid synthase activity | 0.033145 |
| GOTERM_MF_FAT | GO:0019201~nucleotide kinase activity | 0.033145 |
| GOTERM_MF_FAT | GO:0004564~beta-fructofuranosidase activity | 0.033145 |
| GOTERM_MF_FAT | GO:0043169~cation binding | 0.039031 |
| GOTERM_MF_FAT | GO:0016597~amino acid binding | 0.040095 |
| GOTERM_MF_FAT | GO:0008081~phosphoric diester hydrolase activity | 0.040702 |
| GOTERM_MF_FAT | GO:0015198~oligopeptide transporter activity | 0.042025 |
| GOTERM_MF_FAT | GO:0015197~peptide transporter activity | 0.042025 |
| GOTERM_MF_FAT | GO:0035251~UDP-glucosyltransferase activity | 0.044462 |
| GOTERM_MF_FAT | GO:0016667~oxidoreductase activity, acting on sulfur group of donors | 0.044518 |
| GOTERM_MF_FAT | GO:0043167~ion binding | 0.045326 |
| GOTERM_MF_FAT | GO:0046872~metal ion binding | 0.048348 |
| GOTERM_MF_FAT | GO:0016984~ribulose-bisphosphate carboxylase activity | 0.04839 |
| GOTERM_MF_FAT | GO:0004365~glyceraldehyde-3-phosphate dehydrogenase (phosphorylating) activity | 0.04839 |
| GOTERM_MF_FAT | GO:0019842~vitamin binding | 0.049329 |
| GOTERM_MF_FAT | GO:0008289~lipid binding | 0.051255 |
| GOTERM_MF_FAT | GO:0016832~aldehyde-lyase activity | 0.051777 |
| GOTERM_MF_FAT | GO:0005506~iron ion binding | 0.051888 |
| GOTERM_MF_FAT | GO:0019843~rRNA binding | 0.053719 |
| GOTERM_MF_FAT | GO:0020037~heme binding | 0.055459 |
| GOTERM_MF_FAT | GO:0000036~acyl carrier activity | 0.055678 |
| GOTERM_MF_FAT | GO:0016209~antioxidant activity | 0.056174 |
| GOTERM_MF_FAT | GO:0015399~primary active transmembrane transporter activity | 0.056848 |
| GOTERM_MF_FAT | GO:0016701~oxidoreductase activity, acting on single donors with incorporation of molecular oxygen | 0.067612 |
| GOTERM_MF_FAT | GO:0016165~lipoxygenase activity | 0.070261 |
| GOTERM_MF_FAT | GO:0015370~solute:sodium symporter activity | 0.070261 |
| GOTERM_MF_FAT | GO:0016801~hydrolase activity, acting on ether bonds | 0.070261 |
| GOTERM_MF_FAT | GO:0016836~hydro-lyase activity | 0.074306 |
| GOTERM_MF_FAT | GO:0004725~protein tyrosine phosphatase activity | 0.075087 |
| GOTERM_MF_FAT | GO:0015405~P-P-bond-hydrolysis-driven transmembrane transporter activity | 0.077211 |
| GOTERM_MF_FAT | GO:0005200~structural constituent of cytoskeleton | 0.077943 |
| GOTERM_MF_FAT | GO:0046423~allene-oxide cyclase activity | 0.08135 |
| GOTERM_MF_FAT | GO:0031406~carboxylic acid binding | 0.083529 |
| GOTERM_MF_FAT | GO:0004364~glutathione transferase activity | 0.0859 |
| GOTERM_MF_FAT | GO:0005319~lipid transporter activity | 0.09014 |
| GOTERM_MF_FAT | GO:0016780~phosphotransferase activity, for other substituted phosphate groups | 0.091774 |
| GOTERM_MF_FAT | GO:0004784~superoxide dismutase activity | 0.095718 |
| GOTERM_MF_FAT | GO:0016721~oxidoreductase activity, acting on superoxide radicals as acceptor | 0.095718 |
| GOTERM_MF_FAT | GO:0004366~glycerol-3-phosphate O-acyltransferase activity | 0.095718 |
| GOTERM_MF_FAT | GO:0031177~phosphopantetheine binding | 0.095718 |
| GOTERM_MF_FAT | GO:0051287~NAD or NADH binding | 0.097486 |
| GOTERM_MF_FAT | GO:0016860~intramolecular oxidoreductase activity | 0.098937 |
